# TNFα induces Ca^2+^ influx to accelerate extrinsic apoptosis in hepatocellular carcinoma cells

**DOI:** 10.1186/s13046-018-0714-6

**Published:** 2018-03-05

**Authors:** Jianjun Zhu, Mingpeng Jin, Jiaojiao Wang, Hui Zhang, Yousheng Wu, Deyang Li, Xiaoying Ji, Hushan Yang, Chun Yin, Tingting Ren, Jinliang Xing

**Affiliations:** 1 State Key Laboratory of Cancer Biology and Experimental Teaching Center of Basic Medicine, Fourth Military Medical University, 169 Changle West Road, Xi’an, 710032 China; 2 Department of Human Anatomy, Premedical College, North Sichuan Medical College, Nanchong, 637000 China; 3 Department of Pain Treatment, Tangdu Hospital, Fourth Military Medical University, Xian, 710038 China; 4 Division of Population Science, Department of Medical Oncology, Kimmel Cancer Center, Thomas Jefferson University, Philadelphia, PA 19107 USA

**Keywords:** Cell apoptosis, TNFα, Ca^2+^ influx, Hepatocellular carcinoma

## Abstract

**Background:**

Tumor necrosis factor-α has been proven an effective anticancer agent in preclinical studies. However, the translation of TNFα from research to clinic has been blocked by significant systemic toxicity and limited efficacy at maximal tolerated dose, which need urgently to be solved.

**Methods:**

The level of cytosolic Ca^2+^ was assessed by Fura-2 in HCC cells. After changing cytosolic Ca^2+^ level by using agonists or inhibitors, cell apoptosis was detected by flow cytometry. We also detected the effect of ionomycin or parvalbumin on the anti-tumor activity of TNFα in a mice model. Lastly, we studied the roles of cytosolic Ca^2+^ in the mitochondrial-dependent intrinsic apoptosis pathway.

**Results:**

Here, we demonstrated that TNFα induced extracellular Ca^2+^ influx into cytoplasm through transient receptor potential channel in HCC cells. Both cytosolic Ca^2+^ scavenger and Ca^2+^-binding protein PV effectively desensitized hepatocellular carcinoma cells to TNFα, whereas combination ionomycin or 1,4,5-inositol triphosphate significantly sensitized HCC cells to TNFα, indicating that the increased level of cytosolic Ca^2+^ was positively correlated with the TNFα-induced cell apoptosis in vitro. In a nude mice xenograft model, our data revealed that TNFα combined with ionomycin remarkably synergized the anti-tumor effect of TNFα. Furthermore, we found that TNFα-mediated extracellular Ca^2+^ influx accelerated TNFα-induced extrinsic apoptosis through activating calpain/IAP/caspase3 pathway.

**Conclusions:**

Our study provides the evidence supporting a novel mechanism by which TNFα induces extracellular Ca^2+^ influx to enhance cell apoptosis and suggests that increasing the level of cytosolic Ca^2+^ might be an alternative strategy to improve the pro-apoptotic activity of TNFα in HCC cells, although suitable chemical or biological reagents need to be further tested.

**Electronic supplementary material:**

The online version of this article (10.1186/s13046-018-0714-6) contains supplementary material, which is available to authorized users.

## Background

TNFα is a 23KD type II transmembrane protein, which is arranged in stable homotrimers. It is primarily produced by macrophages and a variety of other cells, including NK cells, T lymphocytes, smooth muscle cells, fibroblasts and others [1]. Many preclinical data suggest that TNFα may be used as a highly specific anti-cancer drug against many types of tumors [1, 2]. Moreover, recombinant human TNFα (rhTNFα) has been tested as a systemic treatment of cancer patients in several phase I and phase II clinical trials [3]. However, the initial enthusiasm for the development of TNFα as a systemic treatment has waned when facing significant toxicities and a lack of evidence for therapeutic benefit [2]. How to increase the anti-cancer activity of TNFα at a low-dose condition is the key problem which needs to be solved urgently.

Ca^2+^, as a common signal transduction factor, has played many important roles in the process of cell division, growth, and death [4–6]. The cytosolic Ca^2+^ level is always risen during the process of cell apoptosis [7, 8]. Recent studies have revealed that TNFα is related with remodeling of cytosolic Ca^2+^ homeostasis in a variety of human cells [9, 10]. For example, a study with human pulmonary artery endothelial cells (HPAEC) has reported that TNFα exposure significantly increases TRPC1 expression level and thrombin-induced Ca^2+^ influx in TNFα-stimulated HPAEC is two-fold greater than that in control cells [11]. Moreover, Wang GJ et al. have reported that TNFα induces the increases of cytosolic Ca^2+^ level, CaMKIIδB and CaN expression levels, and thus promotes cardiac hypertrophy [12]. More recently, another study has showed that TNFα induces cell death by the activation of transient receptor potential melastatin (TRPM2) to increase cytosolic Ca^2+^ level, followed by caspase activation and PARP cleavage [13]. Although some studies have reported that the increase of cytosolic Ca^2+^ level induced by TNFα plays important roles in varieties of physical activities, including cell death, it is still largely unknown about the functional roles and mechanisms underlying cytosolic Ca^2+^ in TNFα-induced cell death and whether the remodeling of cytosolic Ca^2+^ facilitates the pro-apoptotic effect of TNFα.

Here, we showed that TNFα induced extracellular Ca^2+^ influx in HCC cells. Importantly, the increased level of cytosolic Ca^2+^ mediated by TNFα was positively correlated with TNFα-induced cell apoptosis. The molecular mechanisms underlying the cytosolic Ca^2+^ in regulating TNFα-induced cell apoptosis were also deeply explored. Furthermore, combination with ionomycin was proven to be able to enhance the anti-cancer activity of TNFα in the mouse model.

## Methods

### Cell culture and public dataset collection

Human cell lines SNU739, SNU368, SNU354, SNU878, JHH-2, Huh-1, HLF, HLE, SMMC772, MHCC97H and QSG-7701 were routinely cultured. The authentication information of cell lines was provided in supplementary files. The detailed information about TCGA database was listed in Additional file 1: Table S3.

### Knockdown, forced expression of target genes

Small interfering RNAs (siRNAs) were synthesized by GenePharma Company (Shanghai, China). The sequences of siRNA and primers were listed in Additional file 1: Table S2.

### qRT-PCR and western blot

RNA extraction, cDNA synthesis and qPCR reactions were performed in Additional file 1: Supplementary Methods. Primers used in this study were list in Additional file 1: Table S2. The western blot assay was performed as described in Additional file 1: Supplementary Methods. The primary antibodies used in this study and the working concentration were listed in Additional file 1: Table S1.

### Measurement of cytosolic Ca^2+^

Cells were loaded with Fura-2/AM (Invitrogen) for 30 min at 37 °C, and examined with a confocal laser scanning microscope FV1000 (Olympus, Tokyo, Japan). After 40 s of baseline recording, TNFα (100 ng/mL) was added where appropriate, and confocal images were recorded every 2 s.

### Cell apoptosis assay

Cell apoptosis was measured by Annexin V-FITC detection Kit (BestBio, Shanghai, China) according to the manufacturer’s protocol. The percentages of total apoptotic cells (both early and late) defined as the AnnexinV-FITC-positive fraction were determined.

### TUNEL assay

Terminal deoxynucleotidyl transferase-mediated dUTP nick-end labeling (TUNEL) assay (Roche Applied Science, Rotkreuz, Switzerland) was performed to analyze cell apoptosis in xenograft tissues according to the manufacturer’s protocol. Images of TUNEL/DAPI-stained sections were grabbed by a confocal laser scanning microscope FV1000 (Olympus). The apoptosis ratio was calculated as the percentage of both TUNEL and DAPI-positive nuclei after at least 500 cells were counted.

### Calpain activity assay

Calpain activity was assayed using a fluorometric kit (Abcam, Cambridge, UK) according to the manufacturer’s protocol.

### Nude mice xenograft model

Nude mice xenograft model was used to assess in vivo tumor growth as described in Additional file 1: Supplementary Methods. TNFα (40 μg/Kg) only, or ionomycin (3 mg/Kg) only, or TNFα (40 μg/Kg) combined with ionomycin (3 mg/Kg) were administered by tail vein injection every three days with vehicle (40% (wt/vol.) of 2-hydroxyproplyl-β-cyclodextrin for one month.

### Immunoprecipitation

For immunoprecipitation experiments, total cell protein or synthesized TNFα were incubated with 200 μL protein A beads (BEAVER, Suzhou, China) supplemented with an antibody overnight at 4 °C. After washing the protein A beads, normalized amounts of total lysates or immunoprecipitated samples were analyzed by SDS-PAGE and western blot.

### Detection of mitochondrial membrane potential

Mitochondrial membrane potential was measured using the fluorescence probe TMRM (Invitrogen) according to the manufacturer’s protocol. Cells were incubated with 10 nM TMRM for 10 min at 37 °C in the dark and images were captured by laser confocal microscope (FV1000, Olympus) and analyzed by ImagePro image analysis software (Media Cybernetics, Silver Spring, MD, USA).

### Detection of cytochrome c release

Cytochrome c release was measured by immunofluorescence staining assay. Briefly, cells were incubated with Mito-Tracker Red (1 μM) for 45 min at 37 °C in the dark. After washing the dyes, cell samples were fixed with 4% paraformaldehyde for 30 min, permeabilized with 0.2% Triton X-100 for 10 min, blocked by 2% BSA for 30 min and incubated with Cytochrome c antibody (1:100) overnight at 4 °C. Cell samples were then incubated with fluorophore-conjugated secondary antibody (1:200) and visualized by a confocal laser scanning microscope FV1000 (Olympus).

### Statistical analysis

SPSS 17.0 software (SPSS, Chicago, IL) was used for all statistical analyses and *P* values less than 0.05 was considered to be statistically significant. Unpaired t-tests were used for comparisons between two groups where appropriate after checking for normal distribution and equal variance of the data. One-way ANOVA were used for comparisons among three or more groups. Correlations between measured variables were tested by Spearman’s rank correlation analyses.

## Results

### TNFα induces extracellular Ca^2+^ influx into HCC cells

**Fig. 1:**
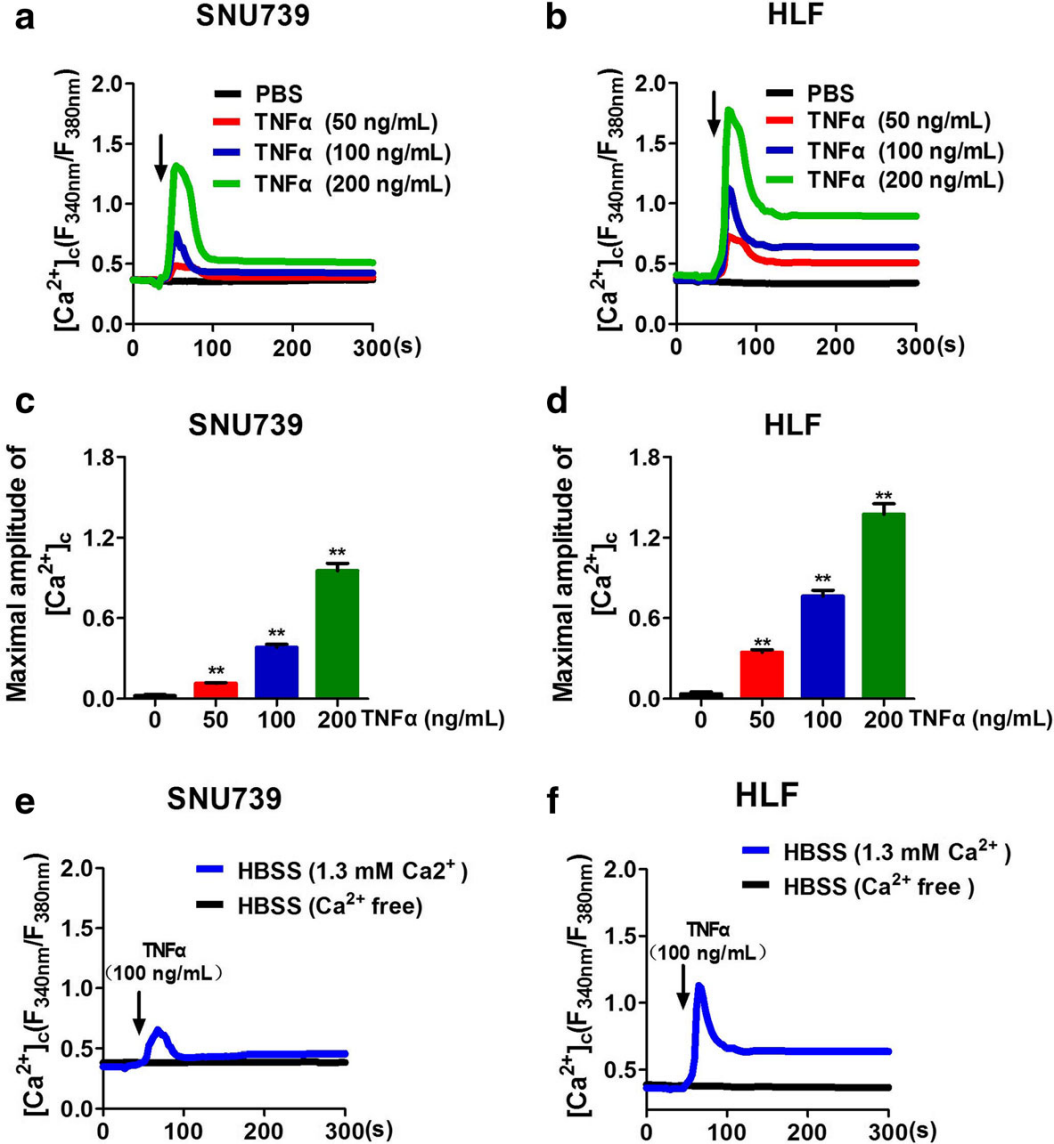
TNFα induces Ca^2+^ influx in HCC cells. **a** and **b** Confocal microscope analysis of [Ca^2+^]_c_ using fluorescent probe Fura-2/AM in SNU739 and HLF cells with treatment as indicated. (Arrow: cells treated with TNFα or PBS)*.* **c** and **d** Quantitative analysis of the maximal increased level of cytosolic Ca^2+^ after TNFα treatment (**d**) and (**f**) Confocal microscope analysis of [Ca^2+^]_c_ in SNU739 and HLF cells with treatment as indicated before stimulation of 100 ng/mL TNFα. HBSS (Ca^2+^ free): cells cultured in Ca^2+^ free-HBSS before TNFα stimulation; HBSS (1.3 mM Ca^2+^): cells cultured in HBSS containing 1.3 mM Ca^2+^ before TNFα stimulation. Data were shown as mean ± SD. All experiments were performed at least three times. * *P* < 0.05; ** *P* < 0.01

We firstly analyzed the level of cytosolic Ca^2+^ after TNFα treatment in SNU739 and HLF HCC cells, and found that fluorescence intensity of cytosolic Ca^2+^ indicator Fura-2 was obviously increased after TNFα treatment, which presented a dose-dependent manner (Fig. 1a-d). In contrast, Fura-2 fluorescence was not changed after TNFα treatment when HCC cells were cultured in calcium-free medium (Fig. 1e, f), which indicated that TNFα induced extracellular Ca^2+^ influx into HCC cells.

### Ca^2+^ influx induced by TNFα is mediated by TRP channel and independent of TNFR

**Fig. 2:**
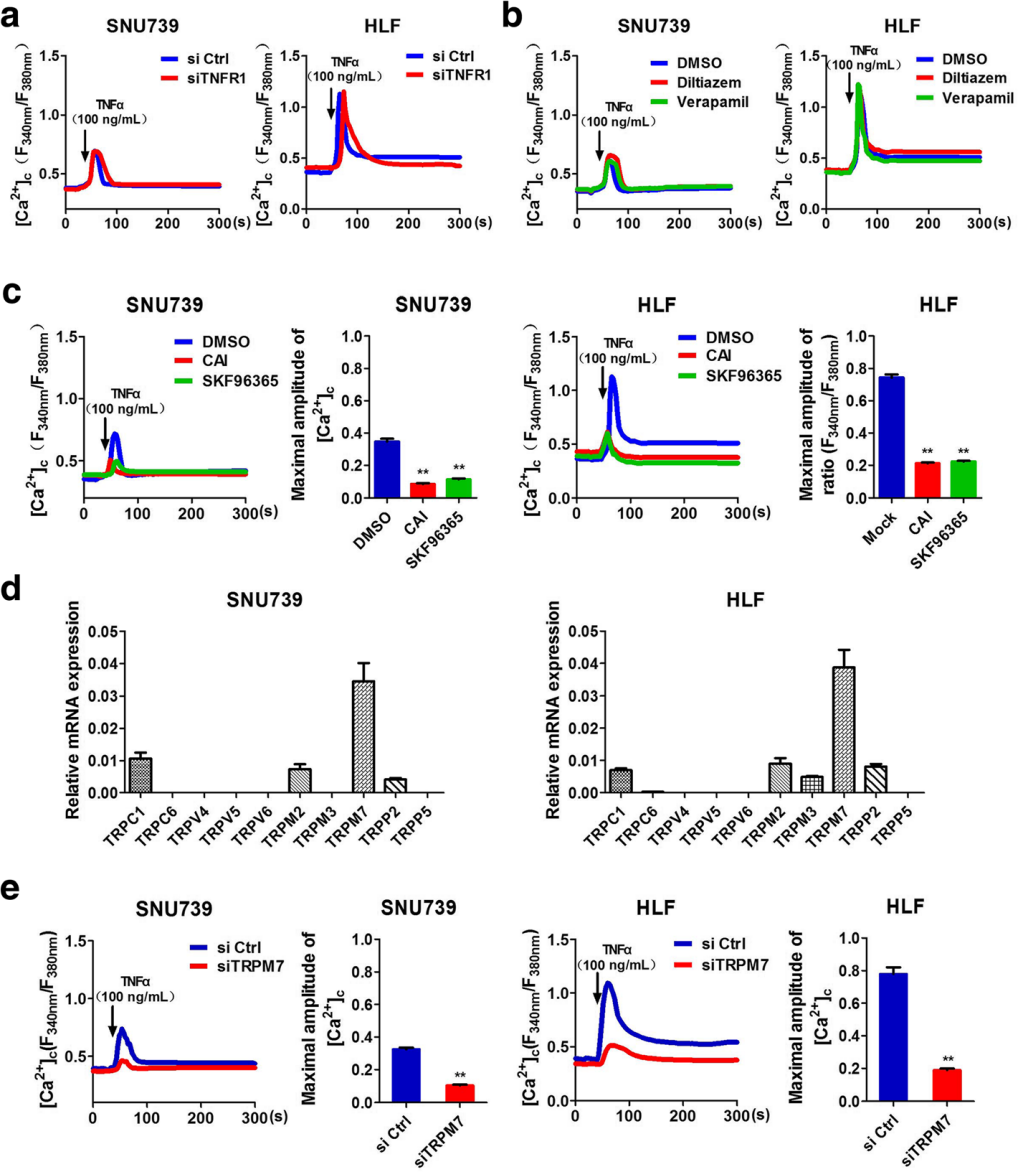
Ca^2+^ influx induced by TNFα was mediated by TRP channels and independent of TNF Receptors. **a** and **e** Confocal microscope analysis of [Ca^2+^]_c_ using fluorescent probe Fura-2/AM in SNU739 and HLF cells with treatment as indicated. siTNFR1: siRNA targeted to TNFR1; siTRPM7: siRNA targeted to TRPM7. **b** and **c** Confocal microscope analysis of [Ca^2+^]_c_ using fluorescent probe Fura-2/AM in SNU739 and HLF cells with treatment as indicated for 30 min before stimulation of 100 ng/mL TNFα. Diltiazem: 10 μM; Verapamil:40 μM; CAI: 10 μM; SKF96365: 100 μM. **d** The relative mRNA expression level of TRP channels in SNU739 and HLF cells. Data were shown as mean ± SD. All experiments were performed at least three times. ** *P* < 0.01

To explore whether tumor necrosis factor receptors (TNFRs) participated in TNFα-mediated Ca^2+^ influx in HCC cells, the expression of TNFR was measured at both the mRNA and protein level by real-time PCR and Western Blot, respectively. We found that TNFR1 but not TNFR2 was expressed in HCC cells (Additional file 2: Figure S1a, b). Moreover, we successfully silenced TNFR1 expression by siRNA in SNU739 and HLF cells, which was verified by real-time PCR and Western Blot. Our data showed that TNFR1 knockdown had no effect on the expression of TNFR2 at both the mRNA and protein levels in HCC cells, suggesting that a compensatory positive regulation of TNFR-2 expression may be excluded. We further confirmed that TRADD, which is a direct downstream molecular of TNFR1 and functions to transfer cell death signal after TNFα stimulation [14], was not able to effectively interact with TNFR1 after siTNFR1 treatment. These data further demonstrated that TNFR1 was successfully knocked down and TNFR1-induced classical extrinsic pathway was inactivated (Additional file 2: Figure S1c-e). Furthermore, our data indicated that the expression level of TNFR1 had no effect on the TNFα-mediated Ca^2+^ influx in HCC cells (Fig. 2a). These results indicate that TNFR pathway is not involved in the process of TNFα-mediated Ca^2+^ influx in HCC cells.

In recently years, 4 kinds of calcium channels in mammal cells have been identified, including voltage-gated calcium channels (VGCC), ligand-gated calcium channels (LGCC), transient receptor potential (TRP), and store-operated calcium channels (SOCE). As LGCCs are only expressed in excitable cells, and our data in Fig. 1e and f also showed that TNFα had no effect on the level of cytosolic Ca^2+^ in cells cultured in calcium-free medium, so the effects of LGCCs and SOCEs on TNFα-mediated Ca^2+^ influx in HCC cells were ruled out. We then explored whether VGCC and TRP channels participated in the process of TNFα-mediated Ca^2+^ influx in HCC cells. Our data showed that the extracellular Ca^2+^ influx in HCC cells was significantly inhibited by both two TRP channel blockers, CAI and SKF96365, whereas the VGCC channel blockers, verapamil and diltiazem had no effect on the TNFα-mediated Ca^2+^ influx in HCC cells (Fig. 2b, c), suggesting that TRP channels may participate in the process of TNFα-mediated Ca^2+^ influx in HCC cells.

It has been reported that a series of TRP channels, including TRPC1, TRPC6, TRPV5, TRPV6, TRPM3, TRPM7, and TRPP5, are implicated in cell apoptosis process [13–15]. Our results demonstrated that TRPM7 was the main channel type expressed in HCC cells (Fig. 2d), which was also demonstrated by bioinformatic analysis based on public transcriptome sequencing data from The Cancer Genome Atlas (TCGA) database (Additional file 2: Figure S1 h). Moreover, we found that TRPM7 knockdown significantly inhibited TNFα-mediated Ca^2+^ influx in HCC cells (Fig. 2e, Additional file 2: Figure S1f, g). Furthermore, co-immunoprecipitation assay showed that TNFα did not directly interact with TRPM7 in SNU739 and HLF cells (Additional file 2: Figure S1i). These findings strongly suggest that TNFα-mediated Ca^2+^ influx may be mediated by TRPM7 and independent of TNFR.

### Decreased cytosolic Ca^2+^ level attenuates TNFα-induced apoptosis

**Fig. 3:**
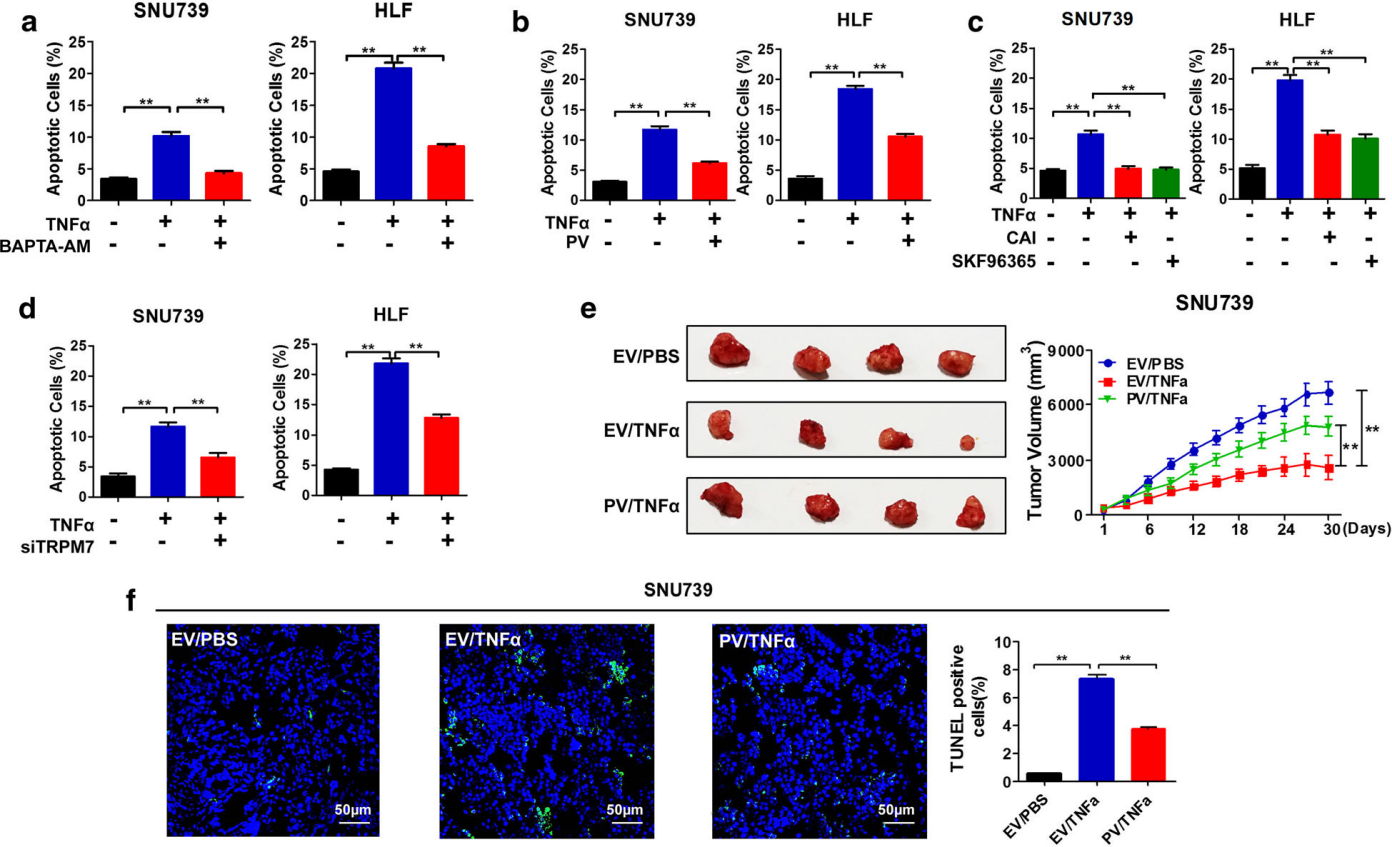
Decreased cytosolic Ca^2+^ level attenuates TNFα-induced apoptosis of HCC cells. **a**-**d** Cell apoptosis analysis by flow cytometry 24 h after treatment as indicated before stimulation of TNFα (100 ng/mL). BAPTA-AM: 10 μM; CAI: 10 μM; SKF96365: 100 μM; PV: expression vector encoding parvalbumin; siTRPM7: siRNA against TRPM7. **e** Tumor growth curves of subcutaneous xenograft tumor model developed from stable HCC cell lines (Right); dissected tumors from sacrificed mice (Left) were shown (*n* = 4 for each group). EV: xenografts developed from SNU-739 cells transfected with empty vector; PV, xenografts developed from SNU-739 cells stably forced expressing parvalbumin; TNFα: 40 μg/Kg. **f** TUNEL staining in tumor tissues of nude mice xenograft model with treatment as indicated. Blue: DAPI; Green: TUNLE positive nucleus. Quantification of cell death ratios were shown in the right. Scale Bar, 50 μm. Data were shown as mean ± SD. EV: empty vector; PV: parvalbumin. All experiments were performed at least three times. * *P* < 0.05; ** *P* < 0.01

We next investigated the roles of TNFα-mediated Ca^2+^ influx in the apoptosis of HCC cells. As shown in Additional file 3: Figure S2a and c, both cytosolic Ca^2+^ scavenger (BAPTA-AM) and Ca^2+^-binding protein parvalbumin (PV) efficiently reduced the level of [Ca^2+^]_c_ after TNFα treatment in HCC cells. We further found that either Ca^2+^ scavengers or PV reduced the percentage of TNFα-induced apoptotic HCC cells (Fig. 3a, b, Additional file 3: Figure S2b, d). We then investigated whether TRP channels mediated the effects of TNFα-mediated Ca^2+^ influx on cell apoptosis. Our data showed that the apoptotic rates of SNU739 and HLF cells were significantly decreased after treatment of TRP channel blocker, CAI or SKF96365 (Fig. 3c, Additional file 3: Figure S2f), and the same effect was also observed after knockdown of TRPM7 in SNU739 and HLF cells (Fig. 3d, Additional file 3: Figure S2 g). Taken together, our results demonstrate that inhibiting the elevation of TNFα-mediated cytosolic Ca^2+^ attenuates TNFα-induced apoptosis in vitro.

To evaluate the role of TNFα-mediated Ca^2+^ influx in tumor growth in vivo, we established the subcutaneous nude mice model. The mice treated with TNFα exhibited significantly decreased growth capacity than those in the control group (Fig. 3e). Moreover, our results also indicated that the growth capacity of xenograft tumors developed from SNU739-PV cells were significantly enhanced than xenograft tumors developed from SNU739-EV cells with TNFα treatment. These results strongly suggest that PV protein may attenuate TNFα-induced cell apoptosis via buffering the TNFα-mediated Ca^2+^ influx in HCC cells. We further confirmed the effect of PV on TNFα-mediated cell apoptosis in xenograft tumor tissues using TUNEL staining (Fig. 3f). These in vivo data support the evidence that extracellular Ca^2+^ influx contributes to the TNFα-induced HCC cell apoptosis.

### Increased cytosolic Ca^2+^ sensitizes HCC cells to TNFα-induced apoptosis

**Fig. 4:**
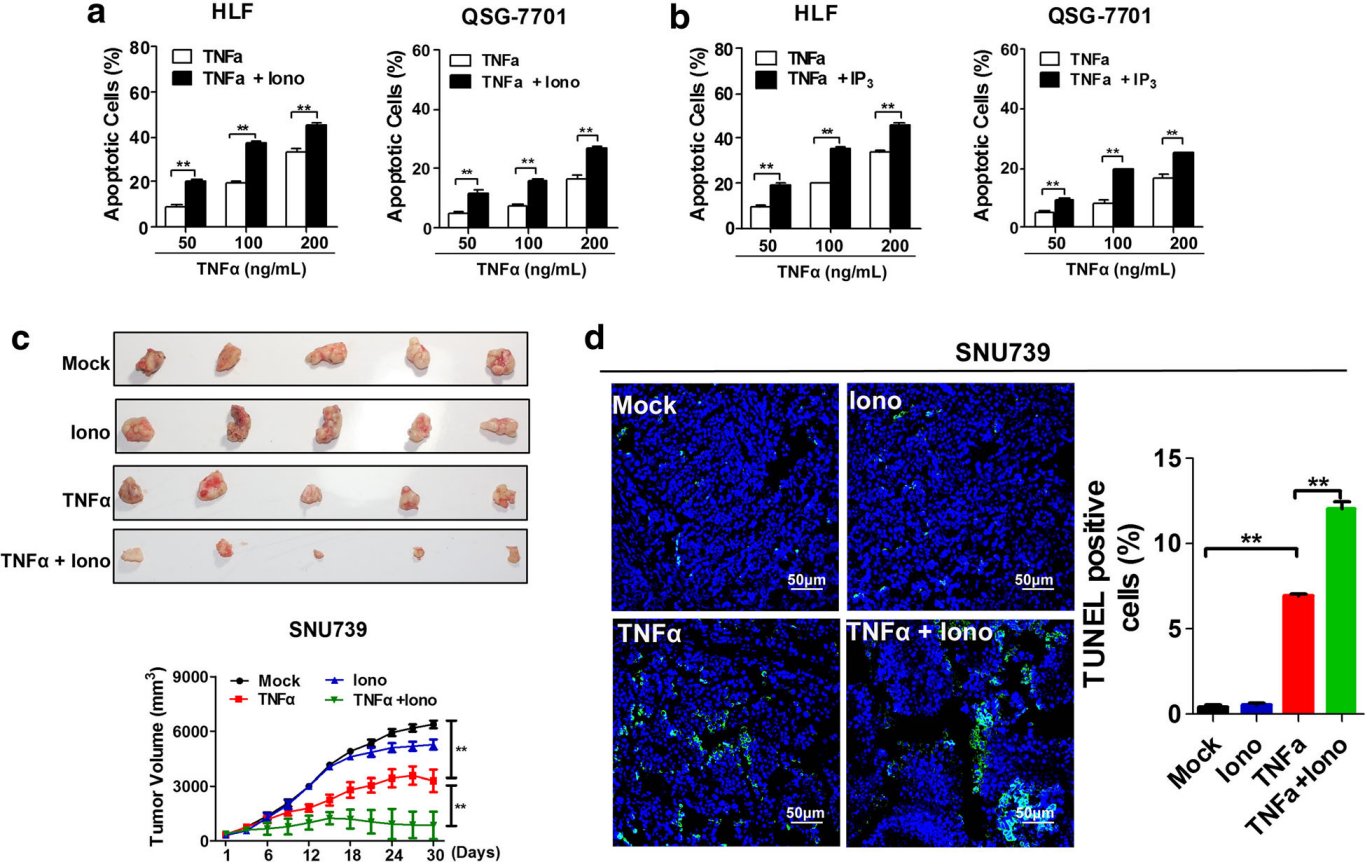
Cytosolic Ca^2+^ sensitized HCC cells to TNFα-induced apoptosis. Apoptosis analysis by flow cytometry 24 h after treatment of different concentration of TNFα with fixed ionomycin (1 μM) (**a**) or IP_3_ (10 μM) (**b**). **c** Tumor growth curves of subcutaneous xenograft tumor model developed from different oncotherapy strategies in SNU739 cells (Lower); dissected tumors (Upper) from sacrificed mice were shown (*n* = 5 for each group). Mock: 40% (wt/vol.) 2-hydroxyproplyl-β-cyclodextrin; TNFα: 40 μg/Kg;Iono: 3 mg/Kg Ionomycin; TNFα + Iono: 40 μg/Kg TNFα and 3 mg/Kg Ionomycin; (**d**) TUNEL staining in tumor tissues of nude mice xenograft model with treatment as indicated. Blue: DAPI; Green: TUNLE positive nucleus. Scale Bar, 50 μm. Data were shown as mean ± SD. All experiments were performed at least three times. * *P* < 0.05; ** *P* < 0.01

Furthermore, we investigated whether increased extracellular Ca^2+^ influx enhanced TNFα-induced apoptosis in HCC cells. Ionomycin (an ionophore with high affinity to calcium) and IP_3_ (an agonist to promote calcium releasing from ER) were used to rapidly elevate [Ca^2+^]_c_ as described previously [16, 17]. As shown in Additional file 4: Figure S3a and d, the optimal concentrations of ionomycin and TNFα were arbitrarily determined as 1 μM and 100 ng/mL, respectively, based on apoptosis rate of less than 10% in normal hepatic cell QSG-7701. Our results showed that ionomycin (1 μM) treatment remarkably increased the level of [Ca^2+^]_c_ in HLF and QSG-7701 cells after TNFα treatment (Additional file 4: Figure S3b). The similar effects were observed in HLF and QSG-7701 cells after IP_3_ (10 μM) treatment (Additional file 4: Figure S3c). Our data further demonstrated that TNFα-induced cell apoptosis was significantly increased at a dose-response manner in HLF and QSG-7701 cells with treatment of either 1 μM ionomycin or 10 μM IP_3_ (Fig. 4a, b, Additional file 4: Figure S3e, f). Taken together, these results indicate that the increased cytosolic Ca^2+^ sensitizes HCC cells and normal hepatocytes to TNFα-induced apoptosis. Moreover, our data indicated that the treatment with a combination of 100 ng/mL TNFα and 1 μM ionomycin exhibited an apoptosis percentage of about 37% in HCC cell HLF, but only about 14% in normal hepatocyte QSG-7701, which is two times as treatment with TNFα (100 ng/mL) only (Additional file 4: Figure S3d, e). We have evaluated the toxicity of TNFα only and the combination of TNFα with ionomycin in normal hepatic cell QSG-7701. Our data showed that treatment with 100 ng/mL TNFα and a combination of 100 ng/mL TNFα and 1 μM ionomycin exhibited an apoptosis percentage of 6.5% and 14.6%, respectively, in normal hepatic cell QSG-7701 (Additional file 4: Figure S3d, e), suggesting that the possible toxicity of TNFα may be increased by a combined use of ionomycin, although the pro-apoptotic effect can be improved.

To evaluate whether ionomycin could sensitize HCC cells to TNFα-induced apoptosis in vivo, we treated xenograft tumors developed from SNU739 cells with ionomycin and TNFα. As shown in Fig. 4c, a significant decrease of growth capacity was observed in xenograft tumors treated with the combination of TNFα (40 μg/kg) and ionomyin (3 mg/kg) for one month when compared with xenograft tumors with treatment of TNFα (40 μg/kg) alone. We further confirmed the effect of combined TNFα and ionomyin treatment on cell apoptosis in xenograft tumor tissues using TUNEL staining (Fig. 4d). These in vivo data further indicated that the increased Ca^2+^ influx sensitizes HCC cells to TNFα-mediated cell apoptosis and the induction of Ca^2+^ influx by ionomycin may synergize the apoptotic effect of TNFα.

### TNFα-induced apoptosis is positively correlated with the level of extracellular calcium influx

**Fig. 5:**
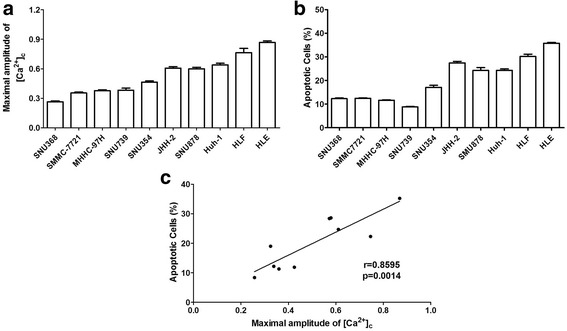
The level of extracellular calcium influx is positively correlated with TNFα-mediated apoptosis. **a** Apoptosis analysis by flow cytometry 24 h after treatment as indicated. **b** Confocal microscope analysis of [Ca^2+^]_c_ level using fluorescent probe Fura-2/AM in 10 kinds of HCC cells with treatment as indicated. **c** Correlational analysis of the maximal increased level of cytosolic Ca^2+^ and apoptotic rates in HCC cells after TNFα (100 ng/mL) treatment. Data were shown as mean ± SD. All experiments were performed at least three times

We then investigated the relationship between TNFα-induced cell apoptosis and the level of extracellular Ca^2+^ influx in 10 HCC cell lines with TNFα treatment (Additional file 5: Figure S4a, b). Our results showed a different degree of TNFα-mediated extracellular Ca^2+^ influx in 10 HCC cells, suggesting that TNFα-mediated extracellular Ca^2+^ influx was a common phenomenon in HCC cells (Fig. 5a). Moreover, we found that TNFα-induced cell apoptosis was obviously positively correlated with the level of extracellular Ca^2+^ influx (Fig. 5b, c).

### TNFα-mediated Ca^2+^ influx activates calpain signaling to enhance extrinsic apoptosis

**Fig. 6:**
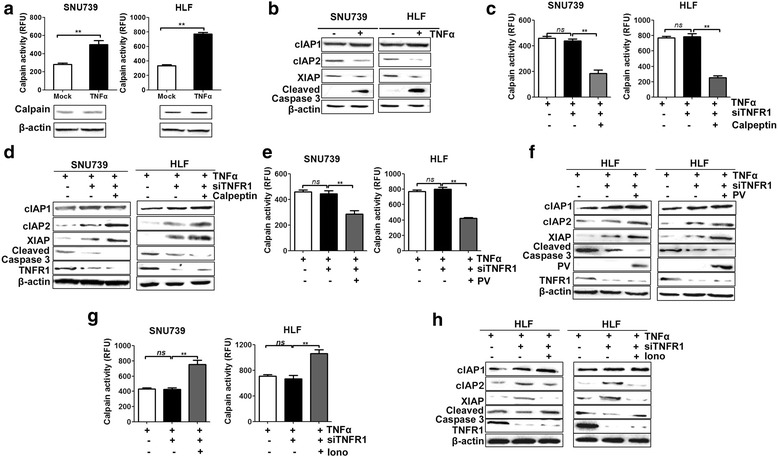
Ca^2+^ influx induced by TNFα activated calpain signaling to enhances extrinsic apoptosis. **a** Western Blot analysis for Calpain expression and analysis of Calpain activity after TNFα (100 ng/mL) stimulation for 4 h in HCC cells with treatment as indicated. **b** Western Blot analysis for expression of cIAP1, cIAP2, XIAP and cleaved caspase-3 after TNFα (100 ng/mL) treatment. **c**–**h** Analysis of Calpain activity and the expression of cIAP1, cIAP2, XIAP and cleaved caspase-3 after treatment with TNFα stimulation for 4 h in HCC cells with treatment as indicated. TNFα: 100 ng/mL; siATNFR1: siRNA target TNFR1; Calpeptin: 40 μM; PV: HCC cells transfected with expression vector encoding parvalbumin, Iono: 1 μM ionomyin. Data were shown as mean ± SD. All experiments were performed at least three times. * *P* < 0.05; ** *P* < 0.01

Previous studies have demonstrated that calpain, a class of Ca^2+^-dependent cysteine proteases, and one of its main substrates the inhibitor apoptosis protein (IAP) plays critical roles in TNFα-induced extrinsic apoptotic pathway [18]. Therefore, we investigated the effect of TNFα-mediated extracellular Ca^2+^ influx on calpain/cIAP/caspase3 pathway. Western blot analysis showed that TNFα had no effect on the expression of calpain. However, the enzymatic activity of calpain was significantly increased in HCC cells with TNFα treatment when compared with control group (Fig. 6a). Our data also showed that the expression of cIAP2 and XIAP was significantly decreased by TNFα treatment, whereas the expression of cIAP1 was not affected. In addition, the expression of the cleaved caspase3 was significantly increased by TNFα treatment (Fig. 6b).

To further investigated the TNFR1-independent mechanism, we re-evaluated the effect of TNFα-meditated Ca^2+^ influx on TNFα-mediated cell apoptosis in HCC cells with TNFR1 knockdown. Moreover, the calpain activity was significantly inhibited by calpeptin (40 μM) treatment, whereas TNFR1 knockdown had no effect on the calpain activity, suggesting that the activation of calpain was independent of TNFR1 (Fig. 6c). In contrast, the expression levels of cIAP2 and XIAP were remarkably increased and the cleaved caspase 3 was decreased by TNFR1 knockdown in HCC cells (Fig. 6d). In addition, the suppression of calpain activity by calpeptin significantly increased the expression of cIAP2 and XIAP and decreased the expression of cleaved caspase 3 in HCC cells with TNFR1 knockdown (Fig. 6d). Besides, similar results were found after the decrease of cytosolic [Ca^2+^] through the forced expression of PV (Fig. 6e, f). In contrast, ionomycin treatment, which can cause the increase of cytosolic [Ca^2+^], significantly inhibited the expression of cIAP2 and XIAP and increased the calpain activity and expression of cleaved caspase 3 in HCC cells with TNFR1 knockdown (Fig. 6g, h). Overall, these results clearly show that cytosolic Ca^2+^ participates in the process of TNFα-induced cell apoptosis through calpain/IAP/caspase3 pathway in HCC cells and demonstrate that TNFα-induced cell apoptosis is affected by both the expressions of TNFR1 and the levels of cytosolic Ca^2+^ in HCC cells. Furthermore, we have also investigated the effect of TNFα on the extracellular Ca^2+^ influx, TRPM7 activity, and activation of calpain signaling in normal hepatic cell QSG-7701. As shown in Additional file 6: Figure S5a, the cytosolic Ca^2+^ level was significantly increased after TNFα treatment in QSG-7701 cells. The extracellular Ca^2+^ influx was significantly inhibited after TRPM7 knockdown, indicating that TNFα-mediated Ca^2+^ influx is also dependent on TRPM7 channel in QSG-7701 cells (Additional file 6: Figure S5b, c). In addition, our results also showed that the activity of calpain was significantly increased after TNFα treatment in normal hepatic cells (Additional file 6: Figure S5d).

### TNFα-mediated Ca^2+^ influx has no effect on mitochondria-dependent intrinsic apoptosis

**Fig. 7:**
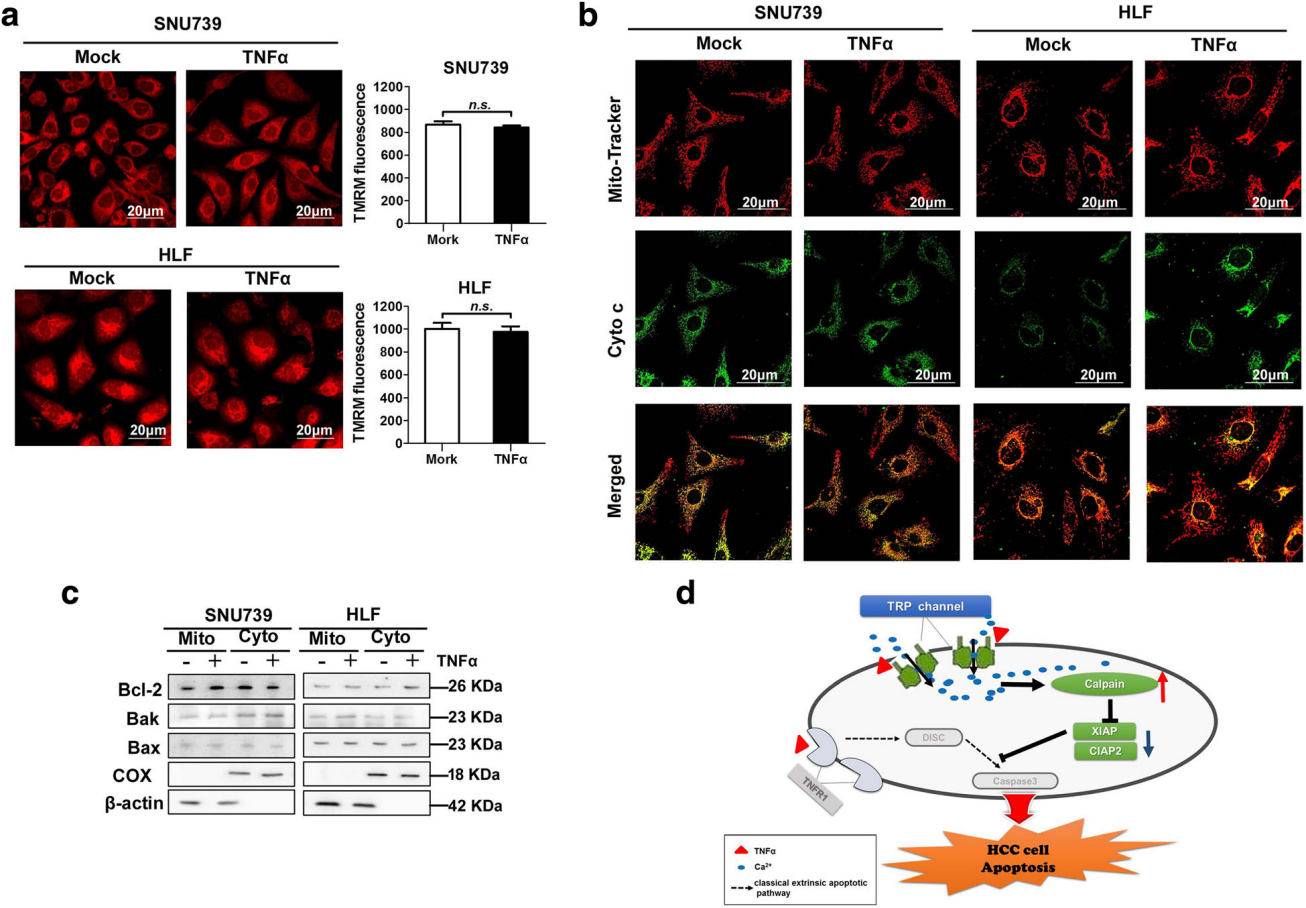
TNFα-induced Ca^2+^ influx had no effect on mitochondria dependent intrinsic apoptosis. **a** Typical confocal microscope images (left) of mitochondrial membrane potential using TMRM fluorescent probe in HCC cells treated as indicated. Quantification analysis of mitochondrial membrane potential were shown in the right. Mock: PBS; TNFα: 100 ng/mL. Scale bar: 20 μm. **b** Cytochrome c release was measured by immunofluorescence in HCC cells with treatment as indicated. Mock: PBS; TNFα: 100 ng/mL. Scale bar: 20 μm. **c** Western blot analysis for expression of Bcl-2, BAX and BAK in cytoplasm and mitochondria of HCC cells with treatment as indicated. β-actin and COX IV were used as loading controls for cytoplasm and mitochondria, respectively. **d** Schematic depicting the cytosolic Ca^2+^ signaling on TNFα-mediated cell apoptosis pathway in HCC cells and underlying mechanics. Data were shown as mean ± SD. All experiments were performed at least three times. * *P* < 0.05; ** *P* < 0.01

To investigate whether TNFα-mediated Ca^2+^influx was associated with mitochondrial-dependent intrinsic apoptosis, we evaluated the mitochondrial membrane potential (MMP), the subcellular location of cytochrome c (Cyto c), and the translocation of Bcl-2 family members in HCC cells after TNFα treatment. As shown in Fig. 7a, TNFα-mediated Ca^2+^ influx had no effect on the MMP of HCC cells. There was also no obvious release of Cyto c from mitochondria to cytoplasm (Fig. 7b), and no obvious translocation of Bax and Bak from the cytoplasm to mitochondria after TNFα treatment (Fig. 7c). Overall, these data indicate that cell apoptosis induced by TNFα-mediated Ca^2+^ influx is independent of intrinsic apoptosis pathway.

## Discussion

TNFα has been proven to be an effective anticancer agent in a series of preclinical studies [2]. However, the promise of systemic TNFα has not translated to patient therapy and the enthusiasm had been cubed due to the toxicity profile and lack of efficacy at maximum tolerated dose (MTD) of the patients [2]. In the past decades, lots of researchers have focused on the modifications of TNFα structure so as to increase its anti-tumor activity, although these modifications are not remarkably effective [1]. Recently, TNFα has been described to be related with levels of cytosolic Ca^2+^ in various cell types [7, 19], although its effect remains largely unclear. In this study, we for the first time demonstrated that TNFα induced extracellular Ca^2+^ influx in HCC cells. Bellomo et al. have reported that TNFα induces a sustained increase in intracellular free Ca^2+^ concentration in mammary adenocarcinoma [19]. Chang et al. have reported that the combined treatment of TNFα and IFN-γ significantly increases the cytosolic Ca^2+^ concentration in pancreatic β cell [7]. However, Carrasquel et al. have reported that TNFα increases the basal level of [Ca^2+^]_c_ after a Ca^2+^ pulse in human sperm [20]. Motagally et al. have reported that the incubation with TNFα decreases depolarization-induced Ca^2+^ influx in postganglionic sympathetic neurons [10]. Moreover, a recent report has provided further supporting evidence, showing that the level of cytosolic Ca^2+^ is decreased in cardiocytes after TNFα treatment [12]. These findings suggest that the level of cytosolic Ca^2+^ is regulated by TNFα, but the dual roles of TNFα in regulating the level of cytosolic Ca^2+^ may be context dependent or cell type specific, which needs more comprehensive investigation.

Furthermore, both TRP channel inhibitor and TRPM7 knockdown significantly inhibited TNFα-mediated Ca^2+^ influx in HCC cells, indicating that the activity of TRPM7 is regulated by TNFα. Our results further showed that TNFα did not directly interact with TRPM7, indicating that TNFα was not a direct activator of TRPM7 channel. Numata et al. have reported that TRPM7 channel is activated by membrane stretch which is produced by negative pressure (3 cm H_2_O) or hypotonic solution [21]. Several organic molecules have also been identified as the activators of TRPM7, including naltriben, mibefradil and bradykinin [22, 23]. Furthermore, Desai et al. have found that TRPM7 channel is activated by caspase-dependent cleavage [24]. However, our results showed that TRPM7 channel was immediately activated upon TNFα stimulation and no direct interaction was observed between TNFα and TRPM7. Therefore, the exact mechanism underlying the activation of TRPM7 channel by TNFα needs to be explored in future study.

Changes in the levels of intracellular Ca^2+^ provide dynamic and highly versatile signals that control kinds of cellular processes, although their importance is perhaps most strikingly exemplified by their functional role in life-and-death decisions [5]. Accumulating evidence have demonstrated the increased levels of cytosolic Ca^2+^ plays a critical role in cell death [8, 19]. Bellomo et al. have reported that the increased intra-nuclear free Ca^2+^ induced by TNFα enhances the activity of Ca^2+^-dependent endonuclease, resulting in DNA fragmentation and cell apoptosis [19]. In consistence with above-mentioned study, we found that the decreased cytosolic Ca^2+^ level attenuates TNFα-induced cell apoptosis, whereas the increased cytosolic Ca^2+^ sensitizes HCC cells to TNFα-induced apoptosis, strongly suggesting that TNFα-induced apoptosis may be positively correlated with the level of extracellular Ca^2+^ influx in HCC cells.

Lots of proteases have been identified as the downstream molecules activated by cytosolic Ca^2+^ to trigger cell death, such as calpain, calcineurin, and DAP kinase [8, 25]. Calpain is a Ca^2+^-activated cysteine protease localized to the cytosol and mitochondria, which has been shown to regulate apoptosis and necrosis [25]. It has been demonstrated that calpain mediates the cisplatin-induced apoptosis in human lung adenocarcinoma cells through truncating Bid to tBid and then inducing the mitochondrial apoptotic pathway [26]. Recently, more and more substrates have been identified to be hydrolyzed specifically by calpain during apoptosis, such as human DNA polymerase epsilon, cain/cabin1, fodrin, p53, caspase 7 and caspase 3 [26–30]. Moreover, inhibitor apoptosis proteins (IAPs) has been found to be hydrolyzed by calpain to promote TNFα-induced classical extrinsic apoptosis [3, 18]. In the present study, we first reported that cytosolic Ca^2+^/calpain/IAPs pathway plays a critical role in synergizing the pro-apoptotic effect of TNFα.

It is generally accepted that mitochondrial Ca^2+^ uptake functions in cell proliferation [5]. However, excessive Ca^2+^ load to the mitochondria may induce apoptosis [8]. Accumulated Ca^2+^ within mitochondria regulates production of ATP, and activates of metabolism-related enzymes involved in cell proliferation [5]. In contrast, mitochondrial Ca^2+^ loading also causes PTP opening to irreversibly commit cells to death by causing IMM depolarization, matrix swelling, release of stored Ca^2+^ and apoptogenic proteins [8]. These findings highlight a dual role of mitochondrial Ca^2+^ in energy provision and induction of cell death, which depend on the amount of mitochondrial Ca^2+^ uptake [31]. Actually, our data showed that MMP, subcellular location of cytochrome c, and the translocation of Bcl-2 family proteins were not obviously changed by TNFα treatment, strongly suggesting that TNFα-induced extracellular Ca^2+^ influx may not trigger mitochondria-dependent cell death in HCC.

Ionomycin is antibiotic produced by *Streptomyces conglobatus*, which is characterized as a calcium ionophore used to increase the cytosolic Ca^2+^ concentration in numerous studies, but the usage of ionomycin in vivo is rare [32]. Recently, Zheng et al. has reported that ionomycin treatment effectively improves the hyperglycaemia and insulin resistance in the mouse model of diabetes [33]. In the present study, our data showed that the combination of TNFα with ionomycin significantly improves the pro-apoptotic effect in HCC cells, although the possible toxicity of TNFα may be also increased. In future study, suitable chemical or biological reagents that specifically increase cytosolic Ca^2+^ need to be further screened and tested for better combination effect with TNFα in HCC treatments.

## Conclusion

In summary, our data provides the strong evidence to support the notion that extracellular Ca^2+^ influx induced by TNFα facilitates the TNFα-mediated extrinsic apoptosis through activating calpain/IAPs/caspase3 signaling pathway and suggests that increasing the level of cytosolic Ca^2+^ might be an alternative strategy to improve the pro-apoptotic activity of TNFα in HCC cells, although suitable chemical or biological reagents need to be further tested.

## Additional files

Additional file 1: Supplemental materials and Methods. **Table S1**. Primary antibodies used for western blot. **Table S2**. Sequence of primers and siRNA. **Table S3**. Public datasets used for bioinformatic analysis. (DOC 97 kb)

Additional file 2: **Figure S1.** (a) and (b) The relative mRNA and protein expression of TNFR1 and TNFR2 measured by qRT-PCR or Western Blot in SNU739 and HLF cells. (c) and (d) qRT-PCR and Western Blot analysis of TNFR1 and TNFR2 mRNA and protein expression levels in SNU739 and HLF cells transfected with siRNA as indicated. (e) TNFR1 protein expression were determined by Co-immunoprecipitation (Co-IP) and western blot in HCC cells as described. (f) and (g) qRT-PCR and western blot analysis of TRPM7 mRNA and protein expression levels in SNU739 and HLF cells transfected with siRNA as indicated. (h) The relative mRNA expression levels of TRPC1, TRPC6, TRPM2, TRPM3, TRPM7, TRPV4, TRPV5, TRPV6, TRPP2, and TRPP5 in HCC tumor tissues were analyzed in public microarray data TCGA downloaded from the Gene Expression Omnibus (GEO) database. (i) The interaction effects between TNFα and TRPM7 were determined by Co-immunoprecipitation (Co-IP) and western blot in HCC cells as described. Data were shown as mean ± SD. All experiments were performed at least three times. * *P* < 0.05; ** *P* < 0.01. (JPEG 945 kb)

Additional file 3: **Figure S2.** Decreased cytosolic Ca2+ level attenuates TNFα-induced apoptosis of HCC cells. (a) Confocal microscope analysis of [Ca^2+^]_c_ level using fluorescent probe Fura-2/AM in SNU739 and HLF cells with treatment as indicated. BAPTA-AM: 10 μM. (b) Cell apoptosis analysis by flow cytometry 24 h after treatment as indicated before TNFα (100 ng/mL) stimulation. BAPTA-AM: 10 μM. (c) Confocal microscope analysis of [Ca^2+^]_c_ level using fluorescent probe Fura-2/AM in HCC cells with treatment as indicated. EV: cells transfected with the empty vector; PV-OE: cells stably forced expressing Parvalbumin protein. (d) Apoptosis analysis by flow cytometry 24 h after treatment as indicated. (e) Western Blot analysis for Parvalbumin expression in SNU739 and HLF cells with treatment as indicated. (f) Cell apoptosis analysis by flow cytometry 24 h after treatment as indicated. CAI: 10 μM; SKF96365: 100 μM. (g) Cell apoptosis analysis by flow cytometry 24 h after treatment as indicated. siTRPM7: siRNA against TRPM7; si Ctrl: negative control siRNA. Data were shown as mean ± SD. All experiments were performed at least three times. * *P* < 0.05; ** *P* < 0.01. (ZIP 1986 kb)

Additional file 4: **Figure S3.** Cytosolic Ca^2+^ sensitized HCC cells to TNFα-induced apoptosis. (a) Apoptosis analysis by flow cytometry 24 h after treatment as indicated. (b) and (c) Confocal microscope analysis of [Ca^2+^]_c_ level using fluorescent probe Fura-2/AM in HLF and QSG-7701 cells with treatment as indicated. TNFα (50 ng/mL) + Iono: 50 ng/mL TNFα combined with 1 μM ionomycin, TNFα (100 ng/mL) + Iono: 100 ng/mL TNFα combined with 1 μM ionomycin, TNFα (200 ng/mL) + Iono: 200 ng/mL TNFα combined with 1 μM ionomycin. TNFα (50 ng/mL) + IP_3_: 50 ng/mL TNFα combined with 10 μM IP_3_; TNFα (100 ng/mL) + IP_3_: 100 ng/mL TNFα combined with 10 μM IP_3_; TNFα (200 ng/mL) + IP_3_: 200 ng/mL TNFα combined with 10 μM IP_3_; (d)-(f) Apoptosis analysis by flow cytometry 24 h after treatment as indicated. Data were shown as mean ± SD. All experiments were performed at least three times. * *P* < 0.05; ** *P* < 0.01. (ZIP 2448 kb)

Additional file 5: **Figure S4.** The level of extracellular calcium influx is positively correlated with TNFα-mediated apoptosis. (a) Confocal microscope analysis of [Ca^2+^]_c_ level using fluorescent probe Fura-2/AM in 10 kinds of HCC cells with treatment as indicated. (b) Apoptosis analysis by flow cytometry 24 h after treatment as indicated. All experiments were performed at least three times. (ZIP 2184 kb)

Additional file 6: **Figure S5.** The role of TNFα-mediated Ca^2+^ influx in normal hepatic cells. (a) and (c) Confocal microscope analysis of [Ca^2+^]_c_ level using fluorescent probe Fura-2/AM in QSG-7701 cells with treatment as indicated. TNFα: 100 ng/mL; siTRPM7: siRNA target TRPM7. (b) qRT-PCR and western blot analysis of TRPM7 mRNA and protein expression levels in QSG-7701 cells transfected with siRNA as indicated. (d) Analysis of Calpain activity after TNFα stimulation for 4 h in QSG-7701 cells with treatment as indicated. Data were shown as mean ± SD. All experiments were performed at least three times. * *P* < 0.05; ** *P* < 0.01. (JPEG 664 kb)

## Abbreviations

cIAP1/2: cellular inhibitor apoptosis protein 1/2
Cyto c: cytochrome c
HCC: hepatocellular carcinoma
IAP: inhibitor apoptosis protein
IFN-γ: interferon gammar
IHC: immunohistochemistry
IMM: inner mitochondrial membrane
LGCC: ligand-gated calcium channels
MMP: mitochondrial membrane potential
MTD: maximum tolerated dose
PARP: poly-ADP-ribose polymerase
PTP: permeability transition pore
PV: parvalbumin
SOCE: store-operated calcium channels
TNFR: tumor necrosis factor receptor
TNFα: tumor necrosis factor alpha
TRADD: tumor necrosis factor receptor type 1-associated death domain protein
TRP: transient receptor potential
TRPM7: transient receptor potential melastatin 7
VGCC: voltage-gated calcium channels
XIAP: X-linked inhibitor apoptosis protein
