# Supplementary material for: TNFα induces Ca2+ influx to accelerate extrinsic apoptosis in hepatocellular carcinoma cells
Source: J Exp Clin Cancer Res. 2018 Mar 5;37:43. doi: 10.1186/s13046-018-0714-6 (PMC5838867; doi:10.1186/s13046-018-0714-6)
Supplement: Supplementary file 1 — Supplemental materials and Methods. Table S1. Primary antibodies used for western blot. Table S2. Sequence of primers and siRNA. Table S3. Public datasets used for bioinformatic analysis. (DOC 97 kb) [file 13046_2018_714_MOESM1_ESM.doc]

**TNFα induces Ca^2+^ influx to accelerate extrinsic apoptosis in hepatocellular carcinoma cells**

Jianjun Zhu^1,2^, MingpengJin^1^, Jiaojiao Wang**^1^**, Hui Zhang^3^, Yousheng Wu^1^, Deyang Li^1^, Xiaoying Ji^1^, Hushan Yang^4^, Chun Yin^1^, Tingting Ren^1*^, Jinliang Xing^1*^

**Additional file 1**

**SUPPLEMENTAL MATERIALA and METHODS**

**Antibodies and Reagents**

Antibodies used in this study were listed in Supplementary Table S1. TNFα was purchased from R&D (Minneapolis, MN, USA). Verapamil, Diltiazem, SKF96365, Calpeptin, and Ionomycin were purchased from Abcam (Cambridge, UK). CAI, BAPTA-AM, 2-hydroxyproplyl-β-cyclodextrin and Inositol 1, 4, 5, - triphosphate (IP_3_) were purchased from Sigma-Aldrich (Munich, Germany). DAPI were purchased from Beyotime (Suzhou, China). Horseradish peroxidase-conjugated AffiniPure Goat Anti-Rabbit/Moust IgG antibody was purchaged from proteintech (Wuhan, China). Fluorophore-conjugated secondary antibody was purchaged from Jackson Immunoresearch (West Grove, PA).

**Quantitative reverse transcription PCR (qRT-PCR),**

RNA extraction, complementary DNA synthesis, and qRT-PCR reactions were performed as described previously[1]. Briefly, total RNA was extracted from the cultured HCC cells using the TRIzol regent (Invitrogen). Reverse transcription reaction were performed using the PrimeScript RT Reagent Kit according to the manufacturer’s instructions. For the qPCR assay, SYBR Green PCR Kit (Takara) was used to amplify the target genes. The relative expression (defined as fold change) of the target genes were quantified by using the 2^-△Ct^ method (△Ct= the C_t_ values of the target genes – the C_t_ values of the β-actin). β-actin was used as internal control. Primer sequences used in this study were synthesized by Sangon Biotech (Shanghai, China) and were provided in Supplementary Table S2.

**Western Blot assay**

Western blot analysis was performed as previously described[2]. Briefly, protein samples were loaded into the 10% polyacrylamide gel, and then transferred to PVDF membrane (Invitrogen). After blocking the PVDF membrane, the PVDF membrane was incubated with the primary antibody at 4°C overnight. Then the PVDF membrane was incubated with the horseradish peroxidase-conjugated AffiniPure Goat Anti-Rabbit/Mouse IgG antibody (1:10000) at room temperature for 1 hour. Finally, the protein bands were visualized by using electrochemiluminescence detection system (Clinx Science Instruments, Shanghai, China). The primary antibodies used in this study were provided in Supplementary Table S1.

**Nude mice xenograft model**

32 four-week-old BALB/c nude mice with the average body weight of 18-22g were randomly divided into groups. Xenografts were initiated by subcutaneous injection of 1×10^7^ HCC cells into the back of mice. One week later, TNFα (40μg/Kg) only, or ionomycin (3mg/Kg) only, or TNFα (40μg/Kg) combined with ionomycin (3mg/Kg) were administered by tail vein injection every three days with vehicle (40% (wt/vol.) of 2-hydroxyproplyl-β-cyclodextrin for one month. The tumor volume (mm^3^) was measured every 3 days and calculated with the formula (length ×wide^2^)/2. One month later, the mice were sacrificed and tumor nodule was harvested and photographed. This study was approved by the ethics committee of the Fourth Military Medical University for animal research.

**Table S1. Primary antibodies used for western blot.**

| **Antibody** | **Company (Cat. No.)** | **Working dilutions** |
| --- | --- | --- |
| calpain | Proteintech(10538-1-AP) | WB: 1/1000 |
| cIAP1 | Proteintech(10022-1-AP) | WB: 1/1000 |
| cIAP2 | Proteintech(24304-1-AP) | WB: 1/500 |
| XIAP | Proteintech (10037-1-AP) | WB: 1/1000 |
| Caspase3 | Proteintech (19677-1-AP) | WB: 1/500 |
| Bcl-2 | Proteintech (12789-1-AP) | WB: 1/1000 |
| BAK | Proteintech (14673-1-AP) | WB: 1/1000 |
| BAX | Proteintech (50599-1-AP) | WB: 1/1000 |
| Parvalbumin | Sigma(P 3088) | WB: 1/1000 |
| TNFR1 | Proteintech (21574-1-AP) | WB: 1/1000 IP: 1/50 |
| TNFR2 | Proteintech (19272-1-AP) | WB: 1/500 |
| Cyto c | Proteintech (10093-1-AP) | IF:1/100 |
| COXⅣ | Proteintech (11242-1-AP) | WB: 1/3000 |
| β–actin | Proteintech (20536-1-AP) | WB: 1/3000 |
| TRPM7 | Abcam (ab109438) | WB：1/1000 IP: 1/50 |
| TRADD | Proteintech (15468-1-AP) | WB：1/1000 |
| TNFα | Abcam (ab215188) | WB: 1/1000 IP: 1/50 |

**Table S2. Sequence of primers and siRNA**

| **1. Primers used in q-PCR analysis** | | |
| --- | --- | --- |
| TNFR1 | forward primer | gctgtaccaagtgccacaaa |
|  | reverse primer | ctccacctgacccatttcct |
| TNFR2 | forward primer | gtacacttaccccagccagt |
|  | reverse primer | agtagacccaaggctgtcac |
| β-actin | forward primer | actcttccagccttccttcc |
|  | reverse primer | tctccttctgcatcctgtcg |
| TRPC1 | forward primer | tccattcgttcattggcacc |
|  | reverse primer | agcacaatcacaaccacgac |
| TRPC6 | forward primer | gctcatccaaactgccaaca |
|  | reverse primer | tccacgcattatcttcccca |
| TRPV4 | forward primer | ttgctgacccacaagaaacg |
|  | reverse primer | cacggaagggcgagttaatg |
| TRPV5 | forward primer | gaacagaccccagtgaagga |
|  | reverse primer | ctcgagaatgagtgcggttg |
| TRPV6 | forward primer | ggggctatcatcatcctgct |
|  | reverse primer | ggcctagcatctggaatcct |
| TRPM3 | forward primer | catgcaccgttttctcacca |
|  | reverse primer | gcgtgtagttgcagcgataa |
| TRPM7 | forward primer | tacacctgtgcctccaagag |
|  | reverse primer | acctcctcccatctcctctt |
| TRPM2 | forward primer | tacgtgctcatggtggactt |
|  | reverse primer | cactgaagtacaaggctgcc |
| TRPP2 | forward primer | ggagaccaagaactgaccga |
|  | reverse primer | actcgtctcaccaggacttg |
| TRPP5 | forward primer | tgctgttgtgttttgtggct |
|  | reverse primer | gggacaaggttgatgacagc |
| **2. Primers used in gene cloning** | | |
| Parvalbumin (PV) | forward primer | TATAAGCTTATGACAGACTTGCTGAACGCTGAGGACATC |
|  | reverse primer | GCTGGATCCGCTTTCAGCCACCAGAGTGGAGAATTCGTC |
| **3. siRNA** | | |
| TNFR1 | sense | GGAACCUACUUGUACAAUGACtt |
|  | antisense | GUCAUUGUACAAGUAGGUUCCtt |
| TRPM7 | sense | CCCGAUAUUAUUUUCCACUAtt |
|  | antisense | UAGUGGAAAUAAUAUCGGGtt |

**Table S3. Public datasets used for bioinformatic analysis**

|  | **Platform** | **Probes/Genes** | **HCC Sample No.** | **Patient Ethnicity** | **Etiology** | **Source URL** |
| --- | --- | --- | --- | --- | --- | --- |
| TCGA | Illumina Hiseq/GA | / | 50 | / | HBV/  HCV | http://cancergenome.nih.gov/ |
