# Supplementary figures and images for: TNFα induces Ca2+ influx to accelerate extrinsic apoptosis in hepatocellular carcinoma cells
Source: J Exp Clin Cancer Res. 2018 Mar 5;37:43. doi: 10.1186/s13046-018-0714-6 (PMC5838867; doi:10.1186/s13046-018-0714-6)

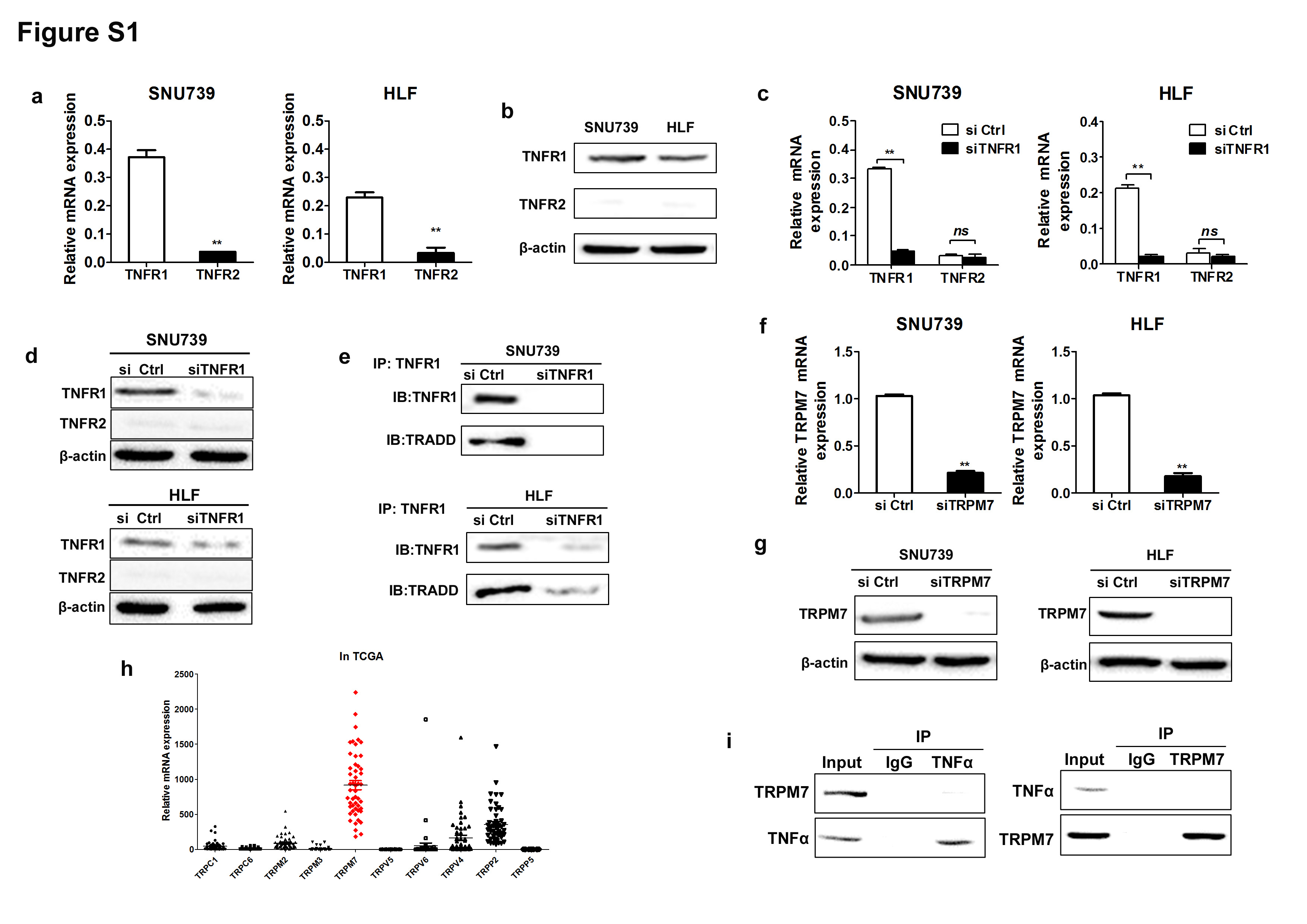

Supplement: Supplementary file 2 — Figure S1. (a) and (b) The relative mRNA and protein expression of TNFR1 and TNFR2 measured by qRT-PCR or Western Blot in SNU739 and HLF cells. (c) and (d) qRT-PCR and Western Blot analysis of TNFR1 and TNFR2 mRNA and protein expression levels in SNU739 and HLF cells transfected with siRNA as indicated. (e) TNFR1 protein expression were determined by Co-immunoprecipitation (Co-IP) and western blot in HCC cells as described. (f) and (g) qRT-PCR and western blot analysis of TRPM7 mRNA and protein expression levels in SNU739 and HLF cells transfected with siRNA as indicated. (h) The relative mRNA expression levels of TRPC1, TRPC6, TRPM2, TRPM3, TRPM7, TRPV4, TRPV5, TRPV6, TRPP2, and TRPP5 in HCC tumor tissues were analyzed in public microarray data TCGA downloaded from the Gene Expression Omnibus (GEO) database. (i) The interaction effects between TNFα and TRPM7 were determined by Co-immunoprecipitation (Co-IP) and western blot in HCC cells as described. Data were shown as mean ± SD. All experiments were performed at least three times. * P < 0.05; ** P < 0.01. (JPEG 945 kb) [file 13046_2018_714_MOESM2_ESM.jpg]

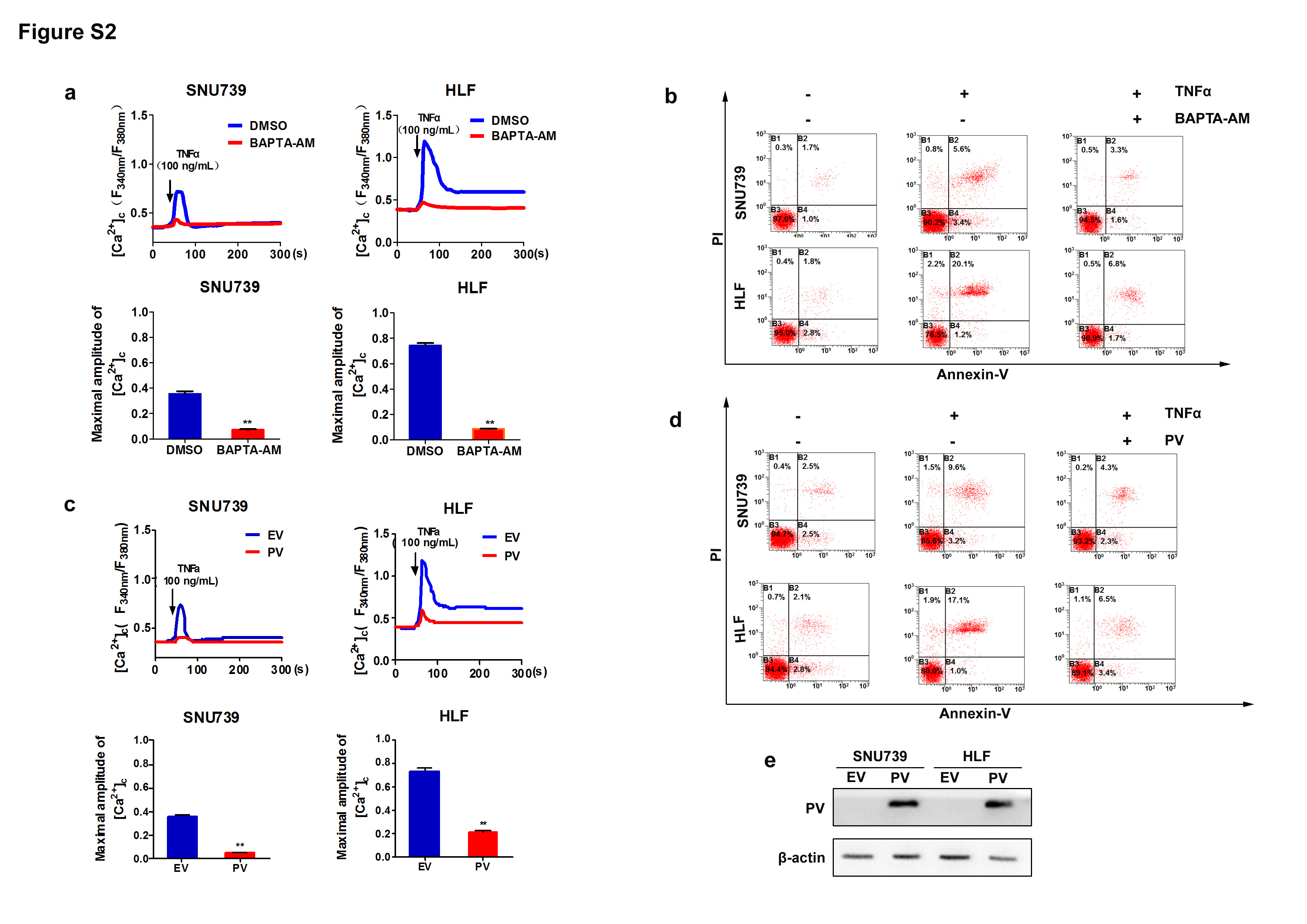

Supplement: Supplementary file 3 — Figure S2. Decreased cytosolic Ca2+ level attenuates TNFα-induced apoptosis of HCC cells. (a) Confocal microscope analysis of [Ca2+]c level using fluorescent probe Fura-2/AM in SNU739 and HLF cells with treatment as indicated. BAPTA-AM: 10 μM. (b) Cell apoptosis analysis by flow cytometry 24 h after treatment as indicated before TNFα (100 ng/mL) stimulation. BAPTA-AM: 10 μM. (c) Confocal microscope analysis of [Ca2+]c level using fluorescent probe Fura-2/AM in HCC cells with treatment as indicated. EV: cells transfected with the empty vector; PV-OE: cells stably forced expressing Parvalbumin protein. (d) Apoptosis analysis by flow cytometry 24 h after treatment as indicated. (e) Western Blot analysis for Parvalbumin expression in SNU739 and HLF cells with treatment as indicated. (f) Cell apoptosis analysis by flow cytometry 24 h after treatment as indicated. CAI: 10 μM; SKF96365: 100 μM. (g) Cell apoptosis analysis by flow cytometry 24 h after treatment as indicated. siTRPM7: siRNA against TRPM7; si Ctrl: negative control siRNA. Data were shown as mean ± SD. All experiments were performed at least three times. * P < 0.05; ** P < 0.01. (ZIP 1986 kb) [file 13046_2018_714_MOESM3_ESM.zip › Figure S2-1.jpg]

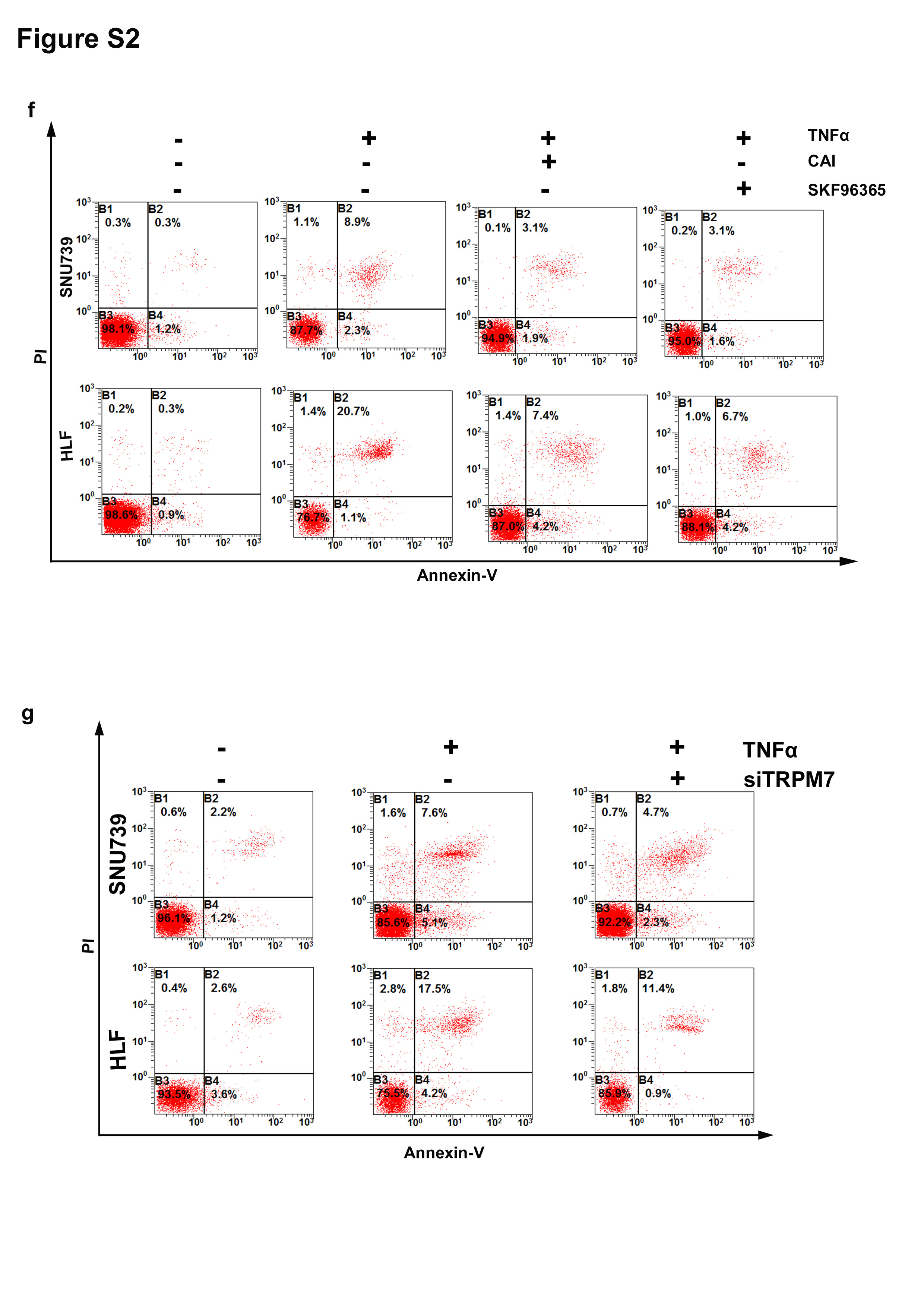

Supplement: Supplementary file 3 — Figure S2. Decreased cytosolic Ca2+ level attenuates TNFα-induced apoptosis of HCC cells. (a) Confocal microscope analysis of [Ca2+]c level using fluorescent probe Fura-2/AM in SNU739 and HLF cells with treatment as indicated. BAPTA-AM: 10 μM. (b) Cell apoptosis analysis by flow cytometry 24 h after treatment as indicated before TNFα (100 ng/mL) stimulation. BAPTA-AM: 10 μM. (c) Confocal microscope analysis of [Ca2+]c level using fluorescent probe Fura-2/AM in HCC cells with treatment as indicated. EV: cells transfected with the empty vector; PV-OE: cells stably forced expressing Parvalbumin protein. (d) Apoptosis analysis by flow cytometry 24 h after treatment as indicated. (e) Western Blot analysis for Parvalbumin expression in SNU739 and HLF cells with treatment as indicated. (f) Cell apoptosis analysis by flow cytometry 24 h after treatment as indicated. CAI: 10 μM; SKF96365: 100 μM. (g) Cell apoptosis analysis by flow cytometry 24 h after treatment as indicated. siTRPM7: siRNA against TRPM7; si Ctrl: negative control siRNA. Data were shown as mean ± SD. All experiments were performed at least three times. * P < 0.05; ** P < 0.01. (ZIP 1986 kb) [file 13046_2018_714_MOESM3_ESM.zip › Figure S2-2.jpg]

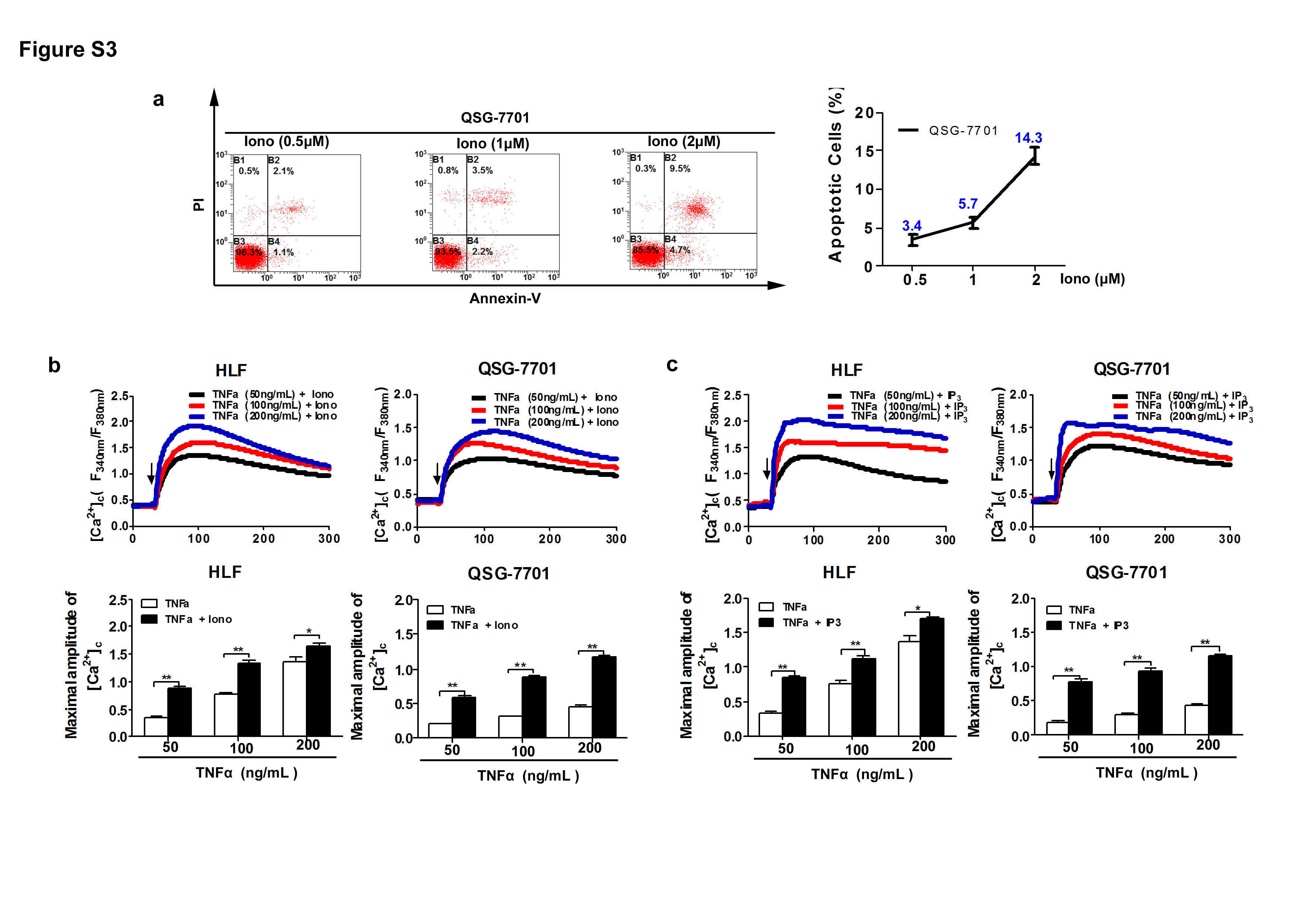

Supplement: Supplementary file 4 — Figure S3. Cytosolic Ca2+ sensitized HCC cells to TNFα-induced apoptosis. (a) Apoptosis analysis by flow cytometry 24 h after treatment as indicated. (b) and (c) Confocal microscope analysis of [Ca2+]c level using fluorescent probe Fura-2/AM in HLF and QSG-7701 cells with treatment as indicated. TNFα (50 ng/mL) + Iono: 50 ng/mL TNFα combined with 1 μM ionomycin, TNFα (100 ng/mL) + Iono: 100 ng/mL TNFα combined with 1 μM ionomycin, TNFα (200 ng/mL) + Iono: 200 ng/mL TNFα combined with 1 μM ionomycin. TNFα (50 ng/mL) + IP3: 50 ng/mL TNFα combined with 10 μM IP3; TNFα (100 ng/mL) + IP3: 100 ng/mL TNFα combined with 10 μM IP3; TNFα (200 ng/mL) + IP3: 200 ng/mL TNFα combined with 10 μM IP3; (d)-(f) Apoptosis analysis by flow cytometry 24 h after treatment as indicated. Data were shown as mean ± SD. All experiments were performed at least three times. * P < 0.05; ** P < 0.01. (ZIP 2448 kb) [file 13046_2018_714_MOESM4_ESM.zip › Figure S3-1.jpg]

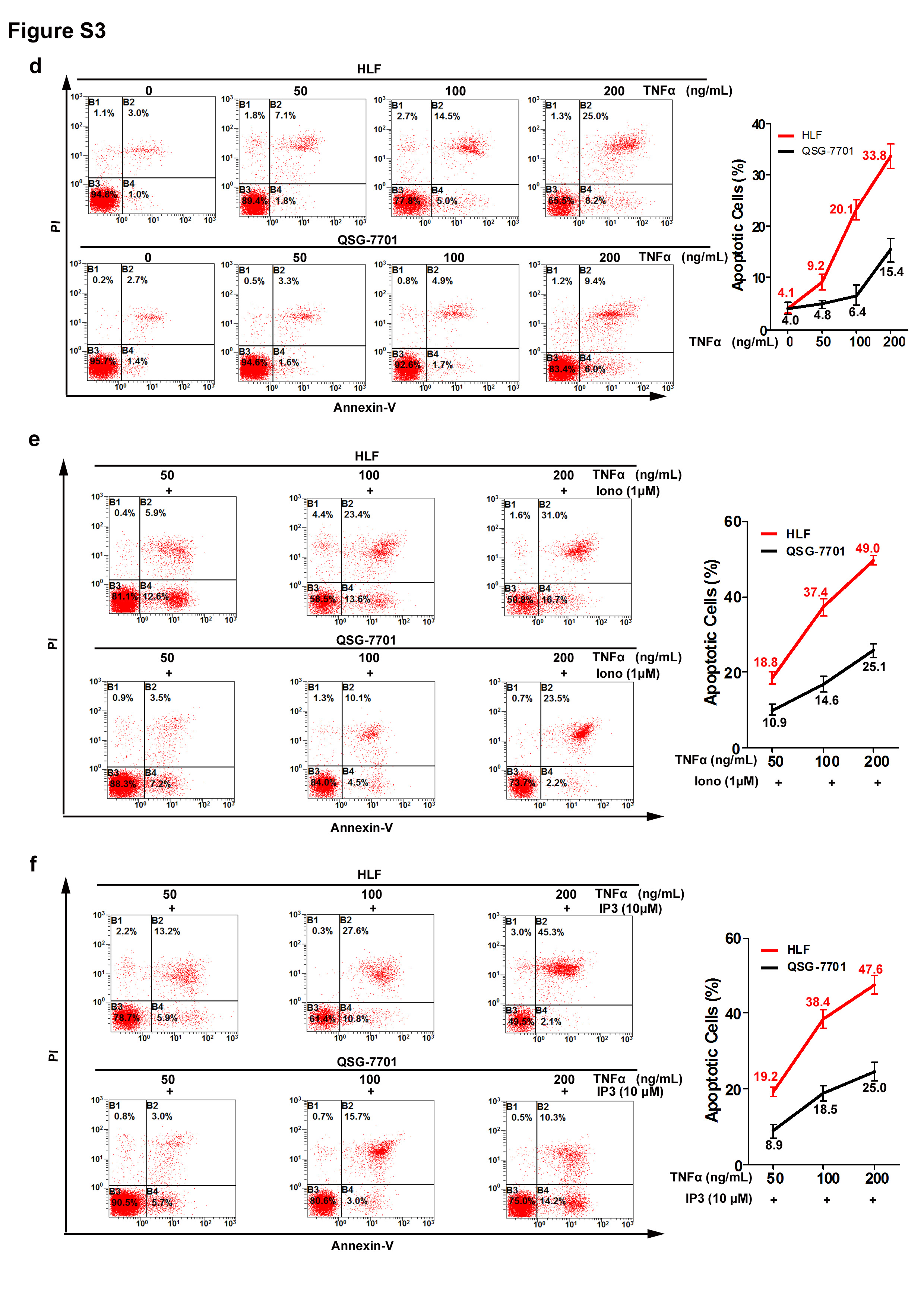

Supplement: Supplementary file 4 — Figure S3. Cytosolic Ca2+ sensitized HCC cells to TNFα-induced apoptosis. (a) Apoptosis analysis by flow cytometry 24 h after treatment as indicated. (b) and (c) Confocal microscope analysis of [Ca2+]c level using fluorescent probe Fura-2/AM in HLF and QSG-7701 cells with treatment as indicated. TNFα (50 ng/mL) + Iono: 50 ng/mL TNFα combined with 1 μM ionomycin, TNFα (100 ng/mL) + Iono: 100 ng/mL TNFα combined with 1 μM ionomycin, TNFα (200 ng/mL) + Iono: 200 ng/mL TNFα combined with 1 μM ionomycin. TNFα (50 ng/mL) + IP3: 50 ng/mL TNFα combined with 10 μM IP3; TNFα (100 ng/mL) + IP3: 100 ng/mL TNFα combined with 10 μM IP3; TNFα (200 ng/mL) + IP3: 200 ng/mL TNFα combined with 10 μM IP3; (d)-(f) Apoptosis analysis by flow cytometry 24 h after treatment as indicated. Data were shown as mean ± SD. All experiments were performed at least three times. * P < 0.05; ** P < 0.01. (ZIP 2448 kb) [file 13046_2018_714_MOESM4_ESM.zip › Figure S3-2.jpg]

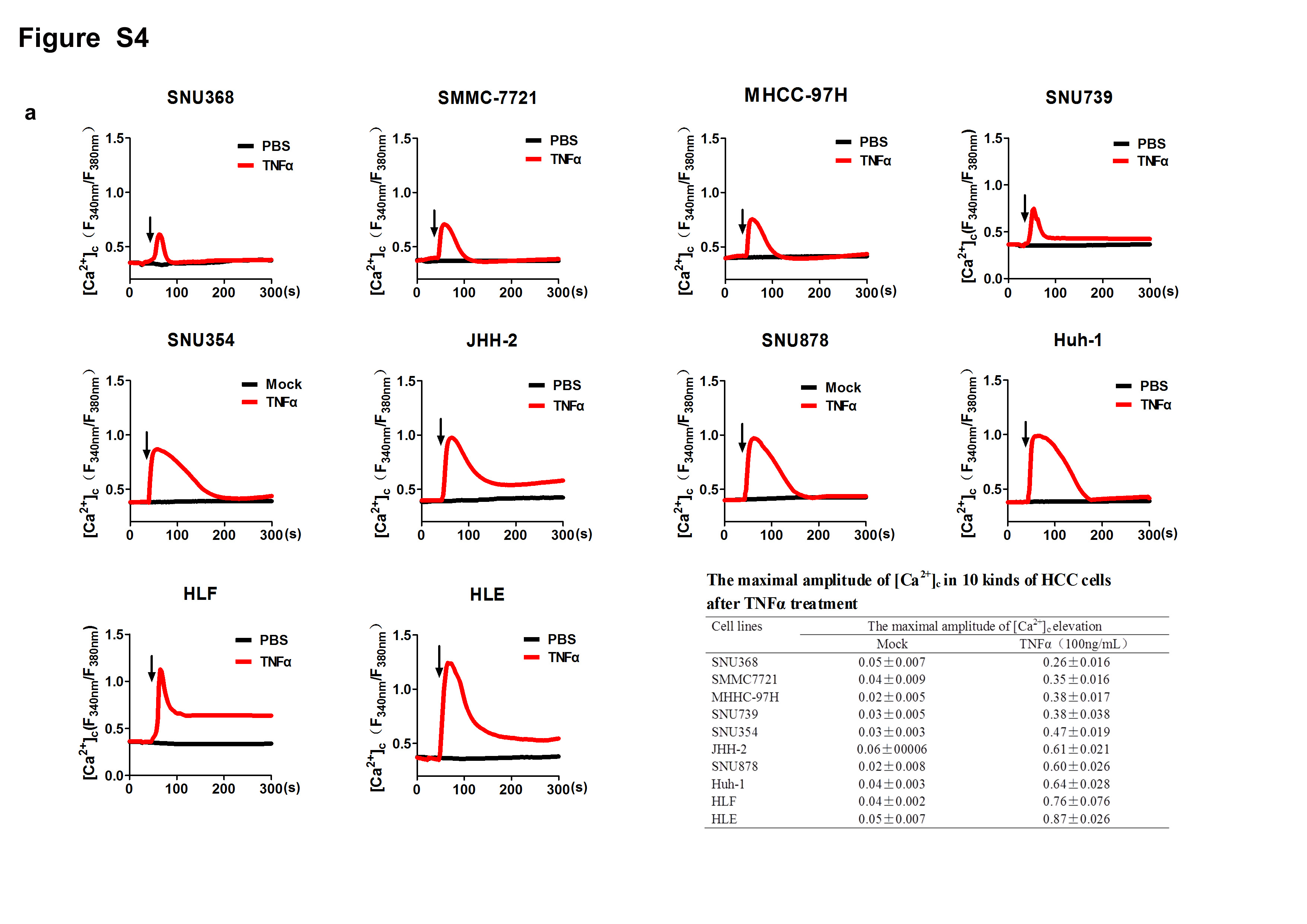

Supplement: Supplementary file 5 — Figure S4. The level of extracellular calcium influx is positively correlated with TNFα-mediated apoptosis. (a) Confocal microscope analysis of [Ca2+]c level using fluorescent probe Fura-2/AM in 10 kinds of HCC cells with treatment as indicated. (b) Apoptosis analysis by flow cytometry 24 h after treatment as indicated. All experiments were performed at least three times. (ZIP 2184 kb) [file 13046_2018_714_MOESM5_ESM.zip › Figure S4-1.jpg]

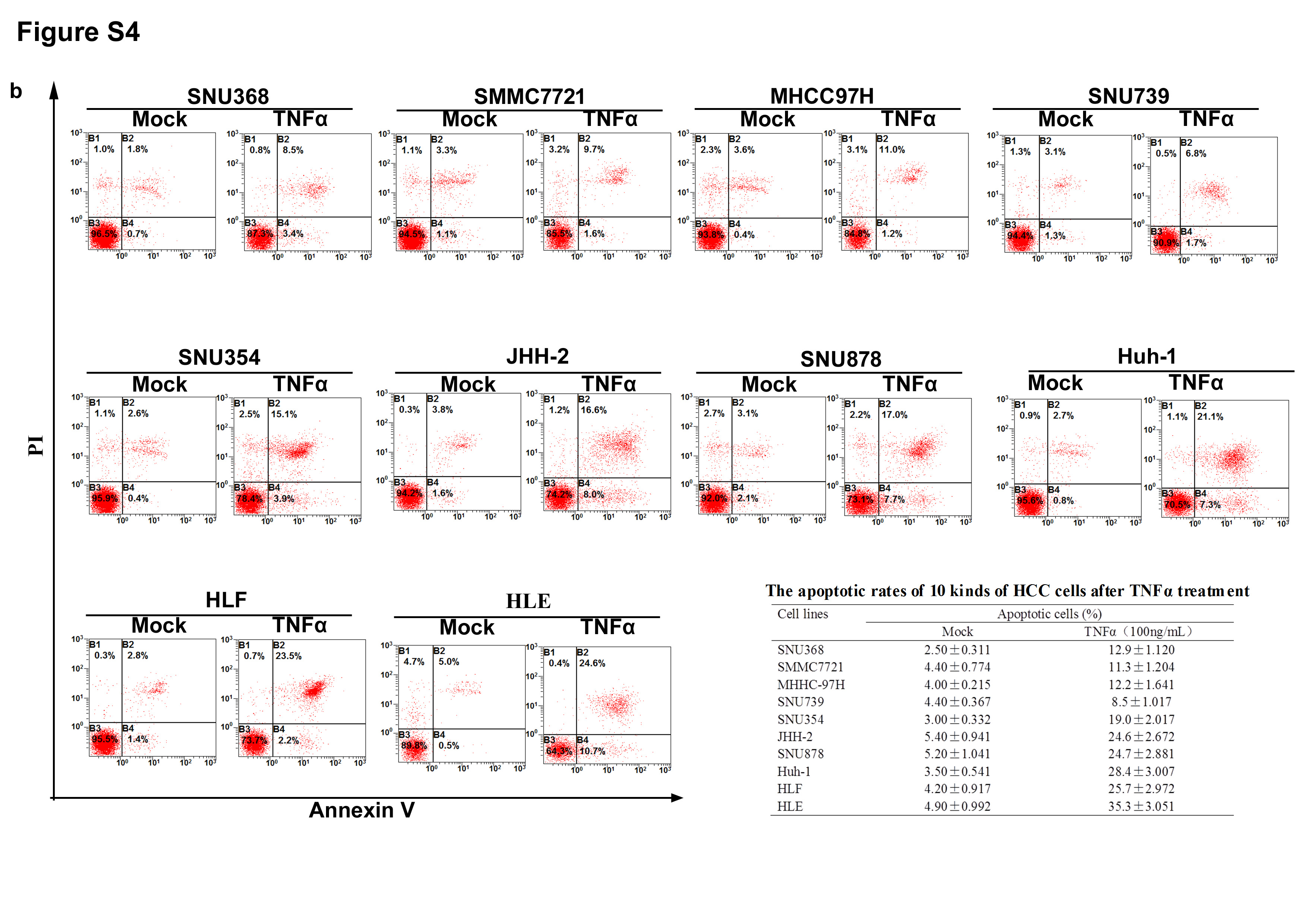

Supplement: Supplementary file 5 — Figure S4. The level of extracellular calcium influx is positively correlated with TNFα-mediated apoptosis. (a) Confocal microscope analysis of [Ca2+]c level using fluorescent probe Fura-2/AM in 10 kinds of HCC cells with treatment as indicated. (b) Apoptosis analysis by flow cytometry 24 h after treatment as indicated. All experiments were performed at least three times. (ZIP 2184 kb) [file 13046_2018_714_MOESM5_ESM.zip › Figure S4-2.jpg]

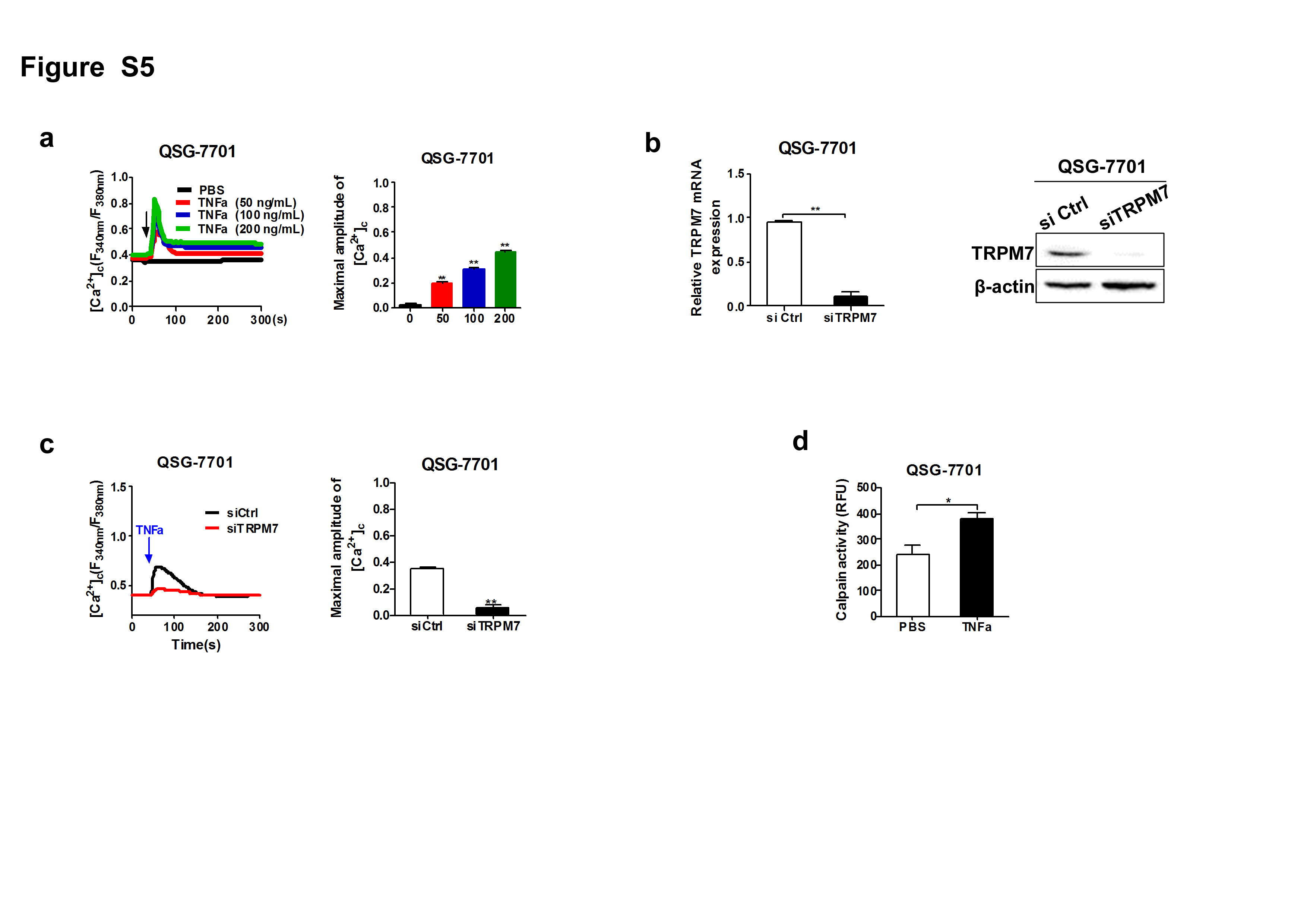

Supplement: Supplementary file 6 — Figure S5. The role of TNFα-mediated Ca2+ influx in normal hepatic cells. (a) and (c) Confocal microscope analysis of [Ca2+]c level using fluorescent probe Fura-2/AM in QSG-7701 cells with treatment as indicated. TNFα: 100 ng/mL; siTRPM7: siRNA target TRPM7. (b) qRT-PCR and western blot analysis of TRPM7 mRNA and protein expression levels in QSG-7701 cells transfected with siRNA as indicated. (d) Analysis of Calpain activity after TNFα stimulation for 4 h in QSG-7701 cells with treatment as indicated. Data were shown as mean ± SD. All experiments were performed at least three times. * P < 0.05; ** P < 0.01. (JPEG 664 kb) [file 13046_2018_714_MOESM6_ESM.jpg]
